# Supplementary material for: HOPE-SIM, a cryo-structured illumination fluorescence microscopy system for accurately targeted cryo-electron tomography
Source: Commun Biol. 2023 Apr 29;6:474. doi: 10.1038/s42003-023-04850-x (PMC10148829; doi:10.1038/s42003-023-04850-x)
Supplement: Supplementary file 2 — Supplementary Information [file 42003_2023_4850_MOESM2_ESM.pdf]

SUPPLEMENTARY INFORMATION

HOPE-SIM, a cryo-structured illumination fluorescence microscopy system for accurately targeted cryo-electron tomography

Shuoguo Li [1,4], Xing Jia [1], Tongxin Niu [1], Xiaoyun Zhang [1], Chen Qi [1], Wei Xu [1], Hongyu Deng [2,4], Fei Sun [1,3,4,*], Gang Ji [1,4,*]

[1] Center for Biological Imaging, Core Facilities for Protein Science, Institute of Biophysics, CAS, Beijing 100101, China.

[2] CAS Key Laboratory of Infection and Immunity, Institute of Biophysics, Chinese Academy of Sciences, Beijing 100101, China.

[3] National Key Laboratory of Biomacromolecules, CAS Center for Excellence in Biomacromolecules, Institute of Biophysics, Chinese Academy of Sciences, Beijing 100101, China.

[4] University of Chinese Academy of Sciences, Beijing 100049, China.

Supplementary Note 1. Points to refine the correlation accuracy between the 3D cryo-SIM and 2D cryo-FIB images.

First, the Pt coating protective layer on the surface of the cryo-sample must be thin enough to be able to identify fiducial markers. Second, we recommend performing fine milling with a smaller beam current of 40~80 pA during the whole cutting process to reduce cutting error caused by the FIB-induced deformation of cryo-lamella. Third, fiducial markers without a complete fluorescence signal or invisible in cryo-FIB images should be preferentially rejected. The standard deviation between cryo-SIM and cryo-FIB images is calculated, which can guide the choice of the number of markers used for correlation. To obtain the best correlation accuracy, there must be sufficient markers around the target. The set of markers with a standard deviation smaller than 200 nm can then be used for further optimization when calculating the correlation.

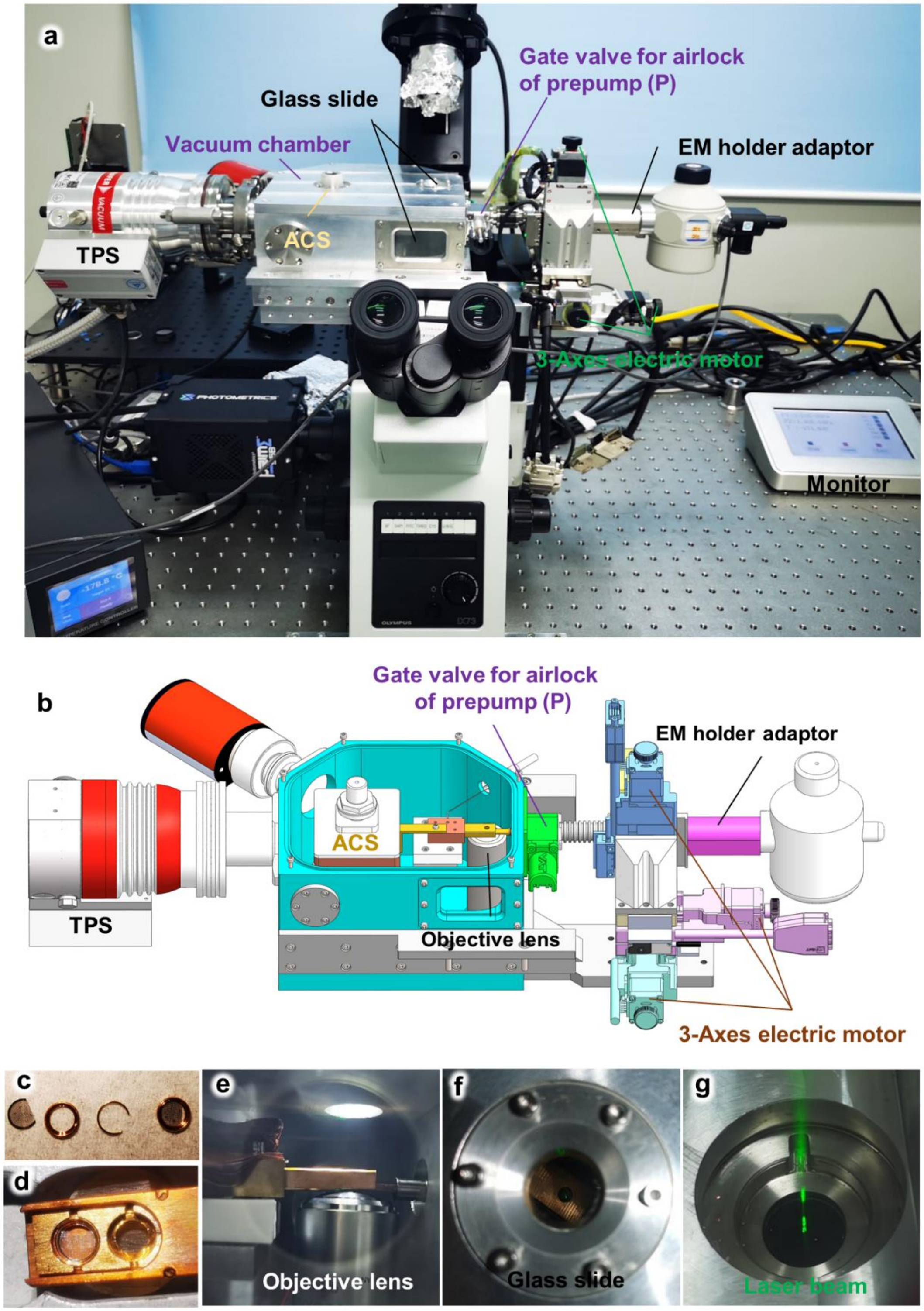

Supplementary Figure 1. **Design and overview of the HOPE-SIM system stage**. (a) A real photograph of an Olympus IX73 inverted microscope with

the mounted HOPE-SIM stage. TPS, turbo pump system; ACS, anti-contamination system. (b) Section view of the design of the HOPE-SIM stage. Each part of the system is labeled and described. (c) D-shape EM finder grid, AutoGrid and C-clip used in the HOPE-SIM system. (d) Real photograph of a custom multiholder tip mounted with AutoGrid in the transfer workstation. (e) Real-time observation of the objective lens and ACS cryo-box under the working conditions via the observation window. (f) Top glass slide of the HOPE-SIM system. (g) Bottom glass window with the laser beam passing through.

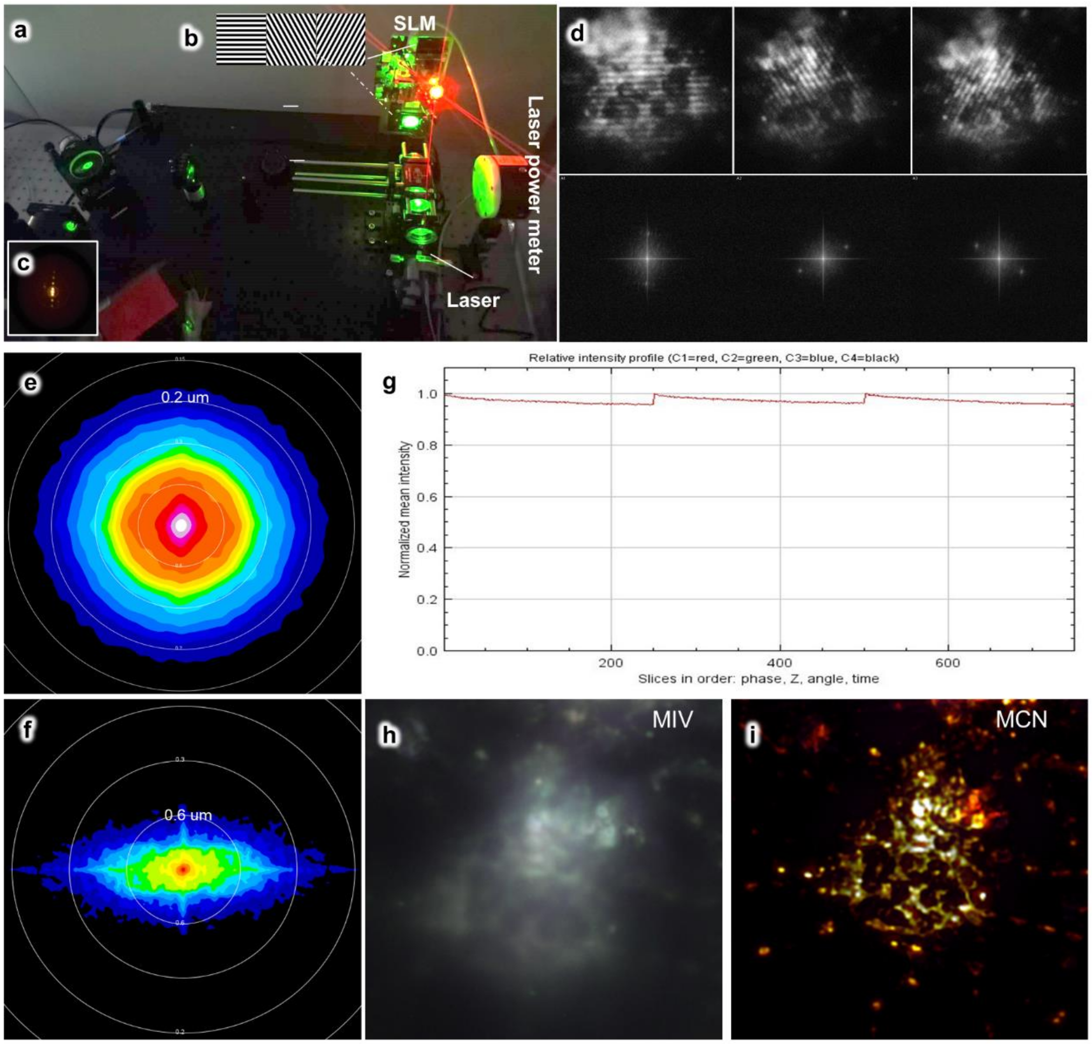

Supplementary Figure 2. **Overview of the HOPE-SIM optical system**. (a) A real photograph of the optical system of HOPE-SIM under working conditions. SLM, spatial light modulator. (b) Three patterned illuminations with different rotations generated by SLM. (c) Diffractions of the order 0 and ±1 are focused on the back focal plane of the objective. (d) Representative raw cryo-SIM images (three illumination patterns) of HeLa cells cultured on the grid and stained with MitoTracker Red CMXRos dye. The corresponding Fourier transformation is shown below with the diffraction points on the order of 0 and ±1. (e) Lateral Fourier spectra of the reconstructed image to show the

attainable resolution (~200 nm) in the *XY* plane. Red, high intensity. Blue, low intensity. (f) Orthogonal Fourier spectra of the reconstructed image to show the attainable resolution (~500 nm) in the *Z* direction. (g) Channel intensity profile generated by SIM-Check [1] to sequentially show the total fluorescence intensity variation along different phases, *z*-slices and angles at one time point. (h) Motion and illumination variation (MIV) generated by SIM-Check to check the stability of the system during 3D cryo-SIM imaging. The phase-averaged and intensity-normalized images for each angle are merged with different colors. The gray-white appearance of MIV indicates good motion stability during illumination. (i) Modulation contrast-to-noise ratio (MCN) heatmap generated by SIM-Check to check the fluorescent signal distribution.

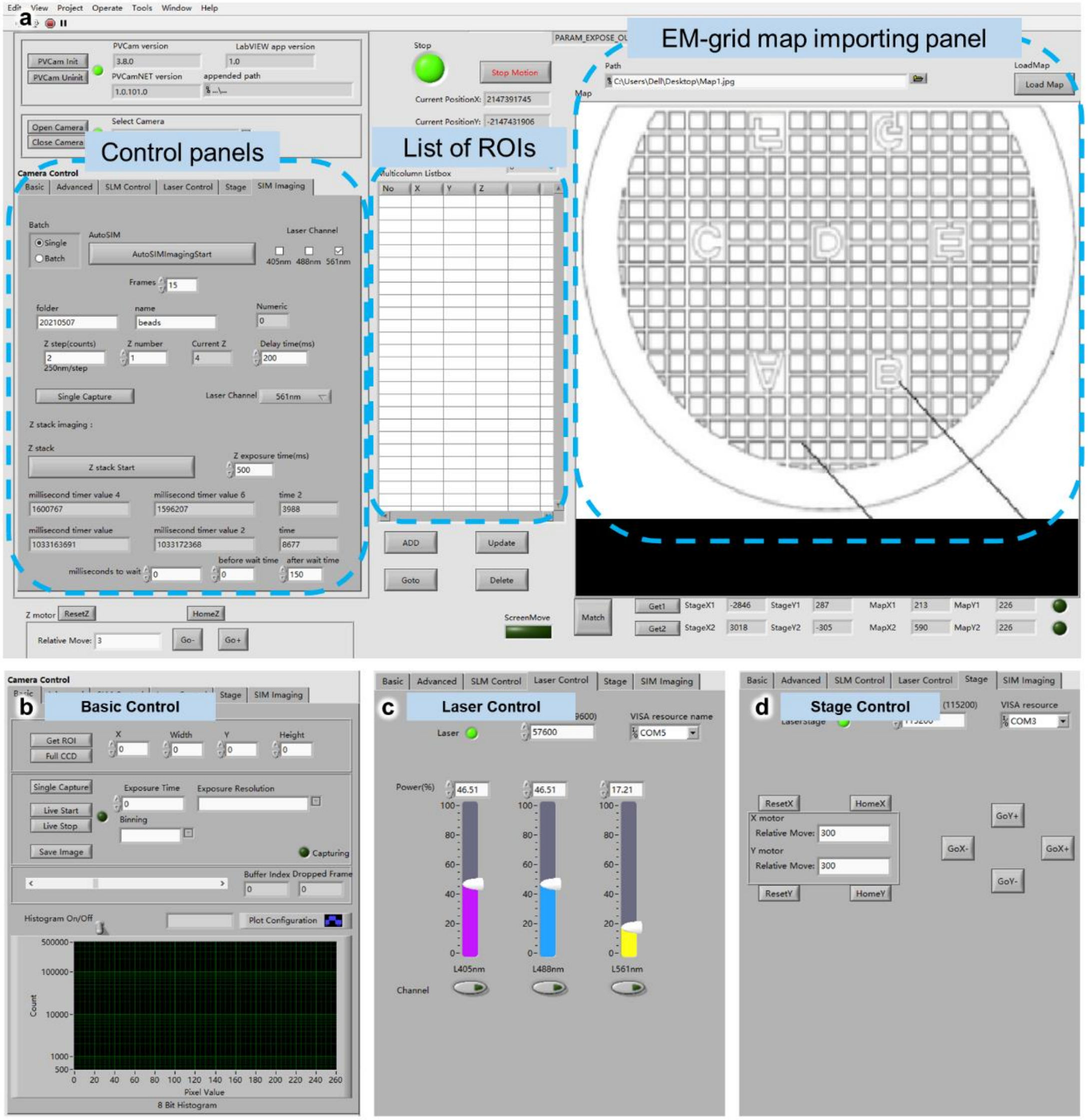

Supplementary Figure 3. **GUI of the HOPE-SIM View software.** (a) The main page of HOPE-SIM View that includes panels for EM-grid map importing, list of ROIs, *z*-focus and microscope control pages. (b) Basic control page. (c) Laser control page. (d) Stage control page.

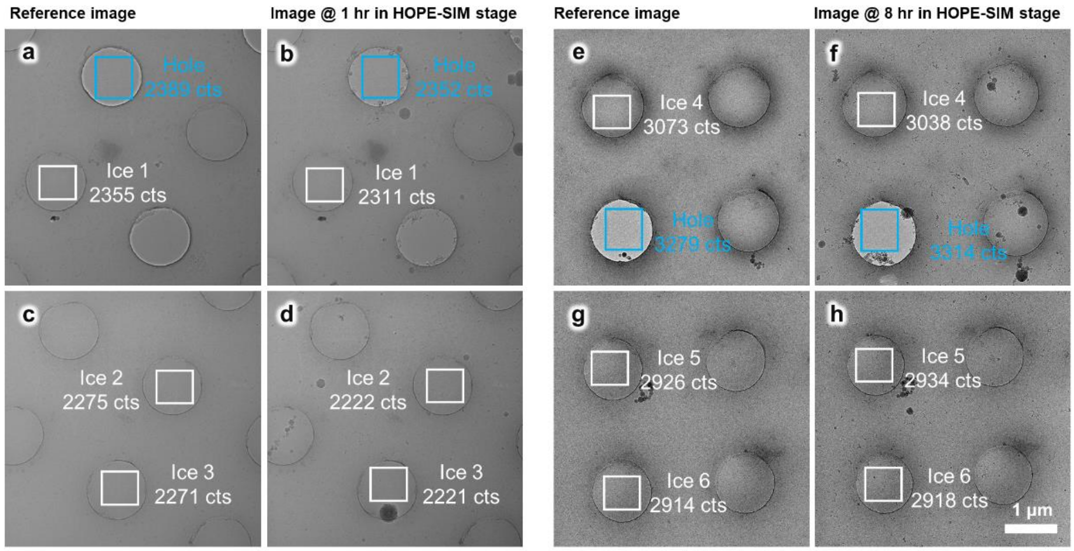

Supplementary Figure 4. Measurement of ice growth rate on the specimen in the vacuum chamber of HOPE-SIM system. Ice growth rate on the specimen gives rise to an increase in scattering contrast in the microscope, which can be measured. We assume that the transmission, defined as the ratio between transmitted and incident electrons, varies over time according to $I_{ice} / I_{hole} = A\exp(-Bt)$, with I_{ice} and I_{hole} the image intensities in areas with and without the ice present, A representing the initial transmission of the substrate, and B representing the scattering of the growing ice layer per unit time. We measured the additional ice scattering using the equation of $[I_{ice}(Ref)-I_{ice}(hr)]/ I_{ice}(Ref)$ to represent the ice growth rate in our system, where $I_{ice}(Ref)$ means the image intensity at the beginning of the test, $I_{ice}(hr)$ means the image intensity after leaving the specimen in the vacuum chamber for a few hours. Before calculating the ice growth rate, $I_{ice}(hr)$ is first normalized with a factor of $I_{hole}(Ref)/I_{hole}(hr)$. The result shows that the averaged ice growth rate in the vacuum chamber of HOPE-SIM system is 0.62% for 1 hr experiment and 1.3% for 8 hrs experiment. In our original HOPE system [2], the measured ice growth rate of the vacuum system was 3.9% per

one hour, therefore the new vacuum system as well as the incorporation of the new anti-contamination system (ACS) of HOPE-SIM has resulted a significantly reduced ice growth rate. To be noted, since the ice growth rate was measured based on cryo-EM micrographs, an additional ice deposition during grid transfer from HOPE/HOPE-SIM to cryo-EM microscope could not be avoided, resulting a potential over-estimation of the ice growth rate. See **Supplementary Data 1** for the source data of the measurement and **Supplementary Data 8** for all the raw cryo-EM micrographs.

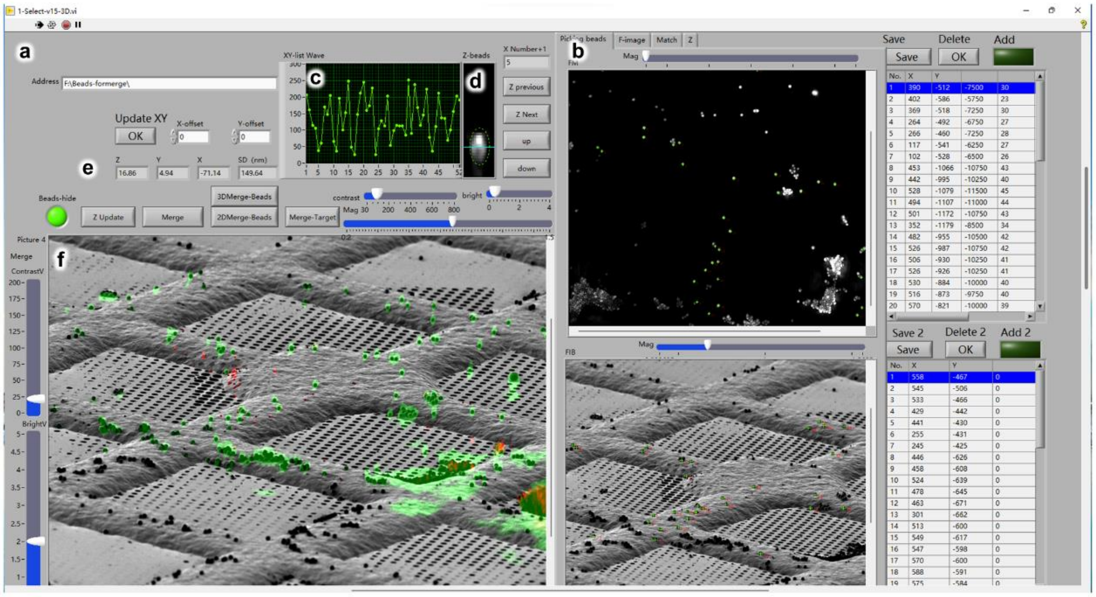

Supplementary Figure 5. GUI of the 3D View software. The main page of the 3D View software that contains the panels to (a) load cryo-FM and cryo-FIB images, (b) pick and list fiducial marker, (c) plot deviations of the correlated fiducial markers, (d) optimize Z-heights of the selected fiducial markers, (e) display the correlation parameters, and (f) show the final merged correlated images.

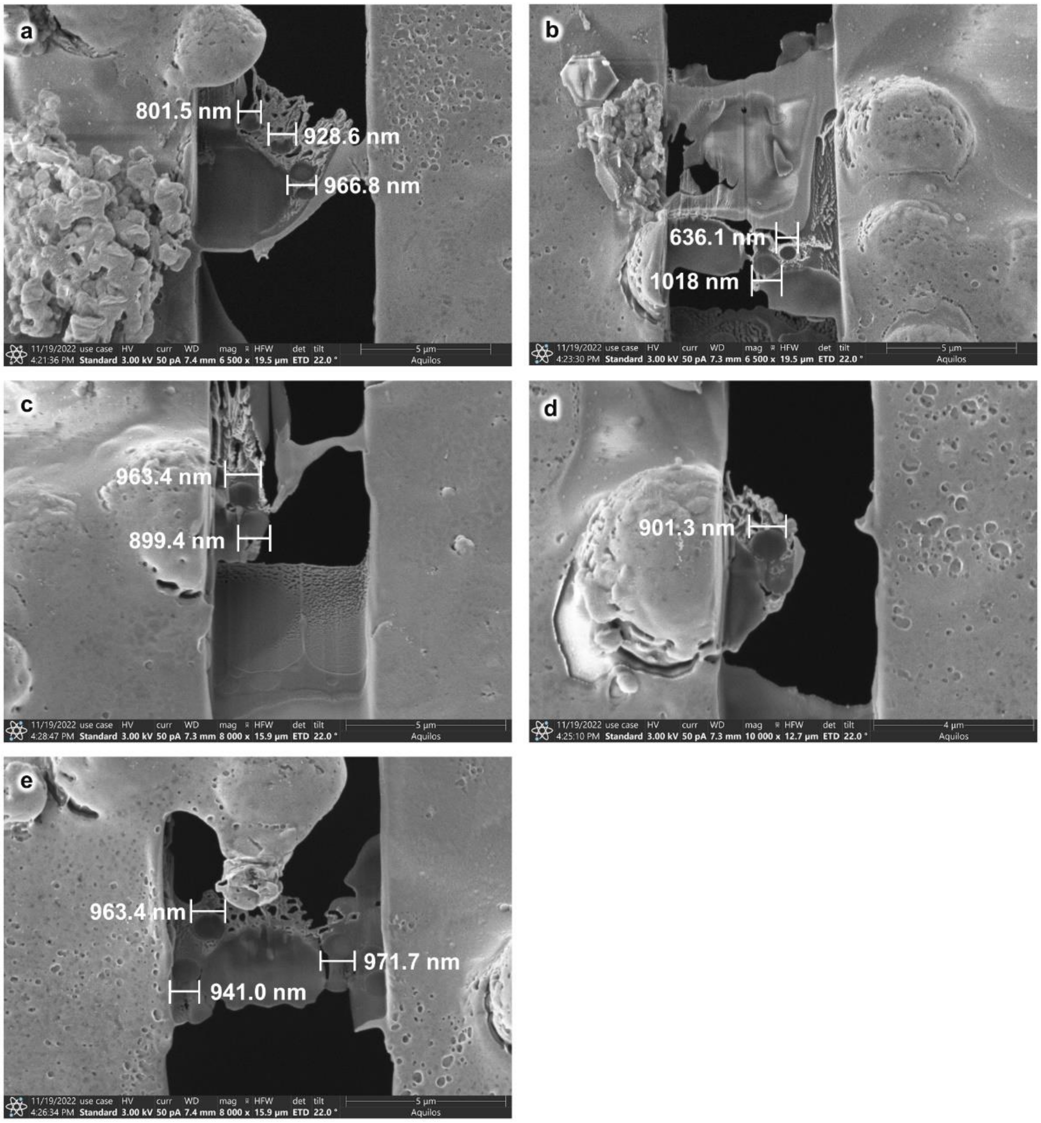

Supplementary Figure 6. List of raw cryo-SEM images of all the cryo-lamellae in Figure 3k. The diameter (nm) of cross-section of each milled microsphere is measured and shown accordingly. The statistics of the measured diameters are shown in **Fig. 3l**.

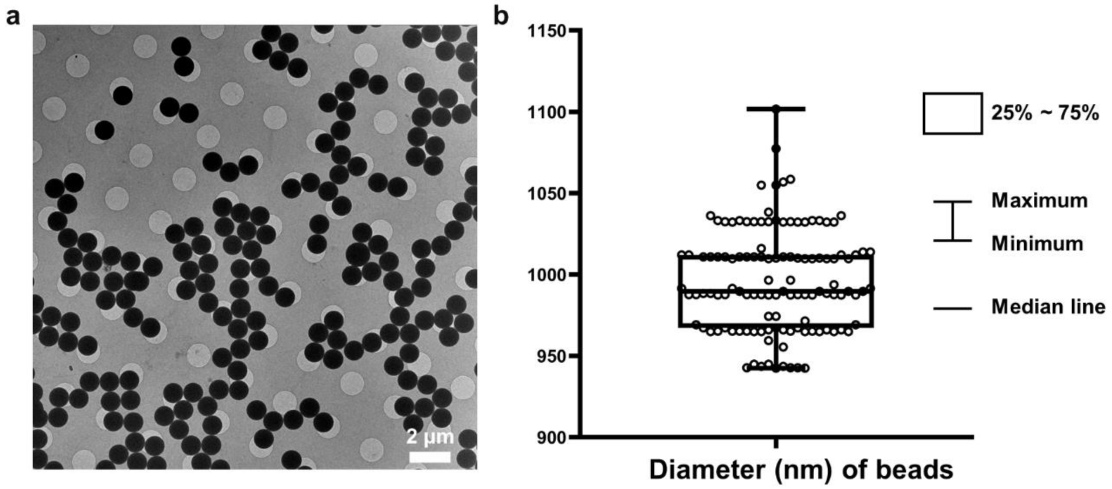

Supplementary Figure 7. Statistical measurement of diameters of blue microspheres by cryo-EM. We measured the diameters of blue microspheres at the liquid nitrogen temperature. To avoid the potential influence of ice contamination for an accurate measurement, the solution of 1 μm blue microspheres were spread onto EM grid and dried. Then the grid was loaded onto a Gatan 626 cryo-holder at the room temperature, which was further loaded into an FEI Talos F200C transmission electron microscope. Then the cryo-holder was cooled by liquid nitrogen for 30 min to reach the cryogenic condition. The high magnification cryo-EM micrograph of microspheres was taken (a) and then their diameters were measured for the subsequent statistics (b). The averaged diameter of the microspheres was 996.5 nm with a maximum of 1101.6 nm and minimum 942.5 nm.

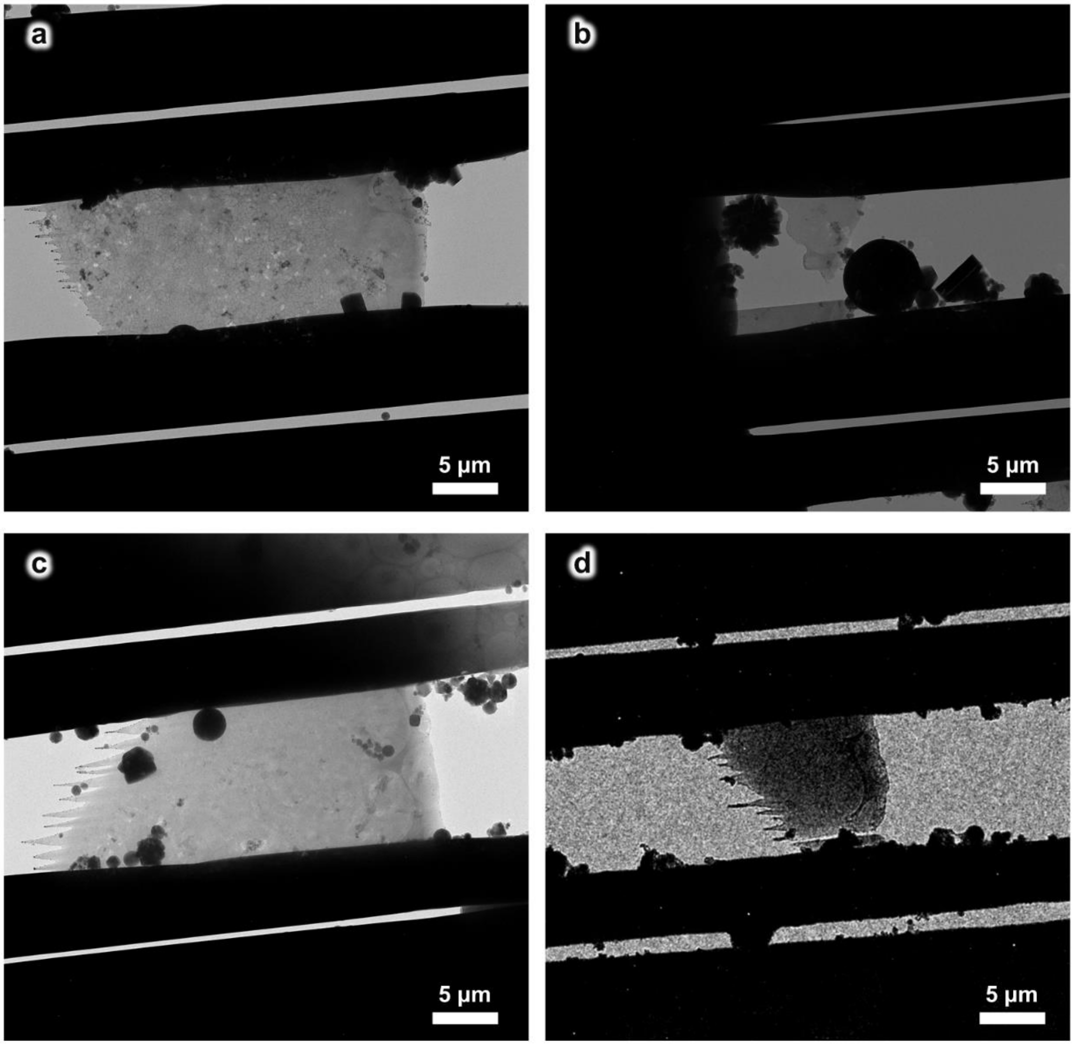

Supplementary Figure 8. Cryo-EM micrographs of cryo-lamellae C1-4 in Figure 5f. To be noted, the C2 cryo-lamella is missing entirely, and the C4 cryo-lamella only retains a small fraction. C1 cryo-lamella showed a critical devitrification not suitable for subsequent cryo-ET study.

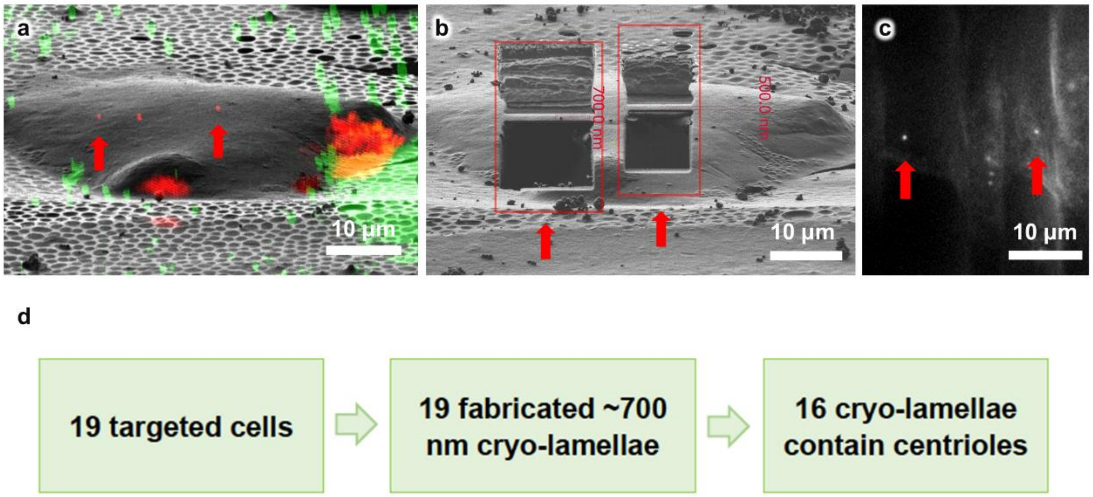

Supplementary Figure 9. Success rate of targeting centrosomes using the HOPE-SIM cryo-CLEM workflow. (a) 3D correlative image indicates the location of the fluorescence (red) -labeled centrosomes of HeLa cells, which are indicated by red arrows. Green, fluorescent microspheres. (b) Site-specific cryo-FIB fabrication was performed to obtain cryo-lamellae (red arrows) with thicknesses of ~700 nm. (c) After cryo-FIB milling, the cryo-EM grid containing the cryo-lamellae was transferred back to the HOPE-SIM system and imaged by cryo-SIM. The fluorescence signals of the target centrosomes were captured (red arrows), proving the success of site-specific cryo-FIB using the HOPE-SIM cryo-CLEM workflow (d). See **Supplementary Data 9** for all the raw images that merged fluorescent signals with bright field images.

Supplementary Table 1. Comparison of characteristics of different non-integrated cryo-CLEM systems

System	Objective lens	Fluorescence resolution	Correlative software	Correlation mode	Applications
**HOPE-SIM**	100X, W.D.2 mm, NA 0.9	~200 nm @ *XY* ~ 500 nm @*Z*	3D View	Cryo-SIM/ cryo-FIB/ cryo-TEM	Mitochondria, viral particles, centrosome in mammalian cells (this study)
**Leica cryo-CLEM**	50X, W.D.0.9 mm, NA 0.9	~ 250 nm @*XY*	SerialEM	Cryo-FM/ cryo-TEM	Virus outside of the cells [3]
**CACM**	100X, W.D.4.1 mm, NA 0.75	~1000 nm @ *Z*	ZEN Connect, module of Zen Blue and SerialEM,	Cryo-Airyscan/ cryo-FIB/ cryo-TEM	Hsp104-GFP in the yeast cells [4]

Supplementary References

1. Ball, G. *et al.* SIMcheck: a Toolbox for Successful Super-resolution Structured Illumination Microscopy. *Sci Rep* **5**, 15915, doi:10.1038/srep15915 (2015).
2. Li, S. *et al.* High-vacuum optical platform for cryo-CLEM (HOPE): A new solution for non-integrated multiscale correlative light and electron microscopy. *J. Struct. Biol.* **201**, 63-75, doi:https://doi.org/10.1016/j.jsb.2017.11.002 (2018).
3. Fu, X. *et al.* AutoCLEM: An Automated Workflow for Correlative Live-Cell Fluorescence Microscopy and Cryo-Electron Tomography. *Sci. Rep.* **9**, 19207, doi:10.1038/s41598-019-55766-8 (2019).
4. Wu, G. H. *et al.* Multi-scale 3D Cryo-Correlative Microscopy for Vitrified Cells. *Structure* **28**, 1231-1237.e1233, doi:10.1016/j.str.2020.07.017 (2020).
