# Supplementary material for: HOPE-SIM, a cryo-structured illumination fluorescence microscopy system for accurately targeted cryo-electron tomography
Source: Commun Biol. 2023 Apr 29;6:474. doi: 10.1038/s42003-023-04850-x (PMC10148829; doi:10.1038/s42003-023-04850-x)
Supplement: Supplementary file 3 — Description of Additional Supplementary Files [file 42003_2023_4850_MOESM3_ESM.pdf]

Description of Additional Supplementary Files

File name: Supplementary Data 1

Description: Source data of transmission loss measurement in Supplementary Figure 4.

File name: Supplementary Data 2

Description: Source data of deviations (nm) of register beads in Fig 3e.

File name: Supplementary Data 3

Description: Source data of measured diameters (nm) of milled beads in Fig. 3k-l.

File name: Supplementary Data 4

Description: Source data of measured diameters (nm) of beads in Supplementary Fig. 7.

File name: Supplementary Data 5

Description: Raw cryo-FIB images of Figs. 3g, h, and j.

File name: Supplementary Data 6

Description: Raw cryo-FIB images of Figs. 4d and e, raw cryo-SEM image of Fig. 4f, and raw cryo-EM image of Fig. 4g.

File name: Supplementary Data 7

Description: Raw cryo-FIB images of Figs. 5d and e, and raw cryo-EM image of Fig. 5f.

File name: Supplementary Data 8

Description: Raw cryo-EM images of Supplementary Fig. 4.

File name: Supplementary Data 9

Description: List of merge images of cryo-lamellae with the thickness of ~700 nm. For each merge image, the fluorescence signal is merged with the wide-field image. The cryo-lamellae are numbered and marked by squared with red for the one containing targeted centrosome and green for the one missing the target.

File name: Supplementary Movie 1

Description: Design and principle of the HOPE-SIM system

File name: Supplementary Movie 2

Description: HOPE-SIM-based cryo-CLEM workflow

File name: Supplementary Movie 3

Description: Aligned cryo-ET tilt series of cryo-lamella containing target MHV-68 viral particles

File name: Supplementary Movie 4

Description: Tomogram of cryo-lamella containing target MHV-68 viral particles

File name: Supplementary Movie 5

Description: Aligned cryo-ET tilt series of cryo-lamella containing target centrosomes

File name: Supplementary Movie 6

Description: Tomogram of cryo-lamella containing target centrosomes

File name: Supplementary Movie 7

Description: Another tomogram of thicker cryo-lamella containing target centrosomes

File name: Supplementary Movie 8

Description: 3D in situ structure of the centriole of HeLa cells rendered on the surface. The triplet tubules are colored yellow, and the internal scaffold is colored pink.
