# Supplementary figures and images for: HOPE-SIM, a cryo-structured illumination fluorescence microscopy system for accurately targeted cryo-electron tomography
Source: Commun Biol. 2023 Apr 29;6:474. doi: 10.1038/s42003-023-04850-x (PMC10148829; doi:10.1038/s42003-023-04850-x)

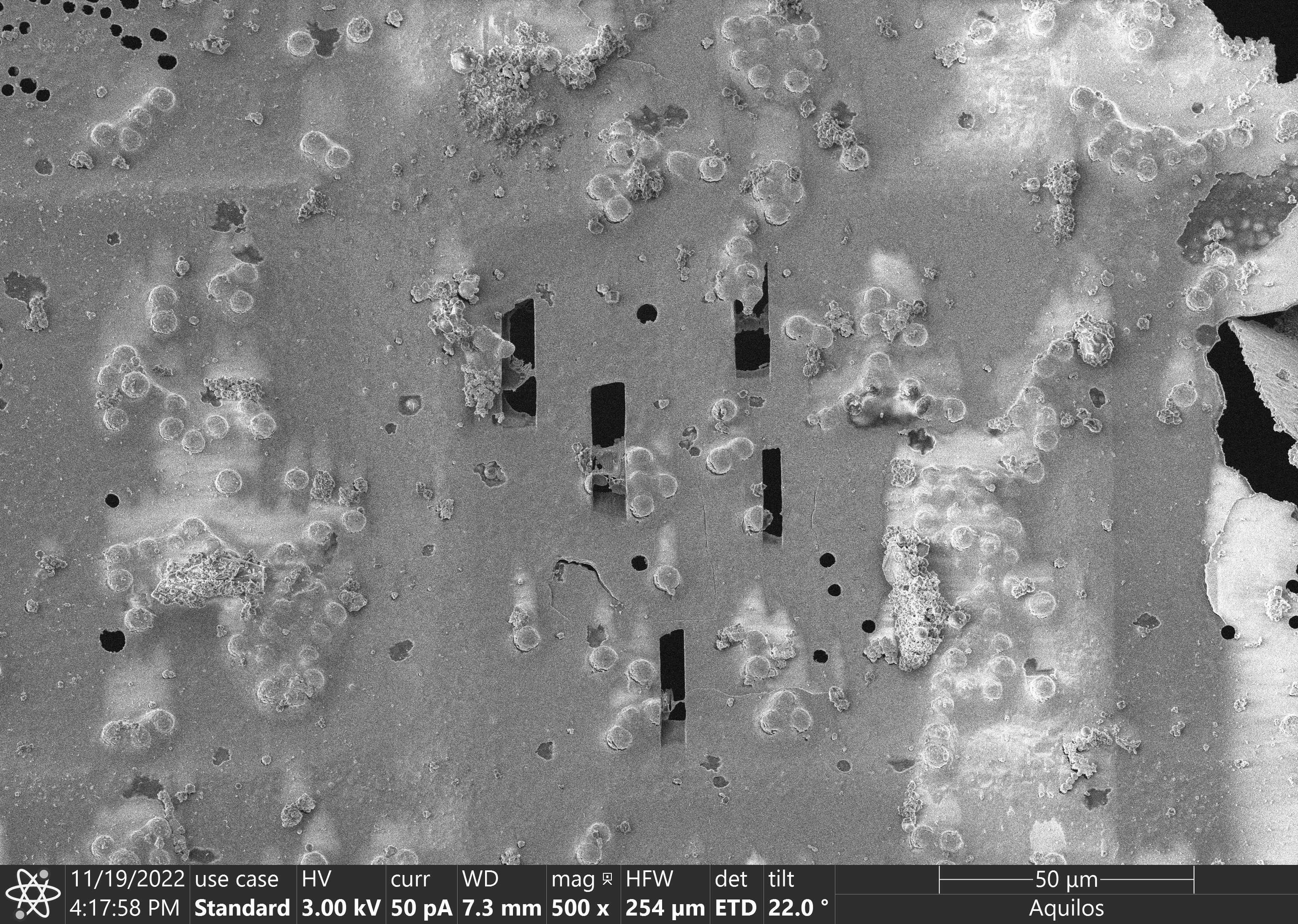

Supplement: Supplementary file 4 — Supplementary Data 1-9 [file 42003_2023_4850_MOESM4_ESM.zip › Supplementary Data 5/raw-cryoFIB-micrograph-Fig.3j.tif]

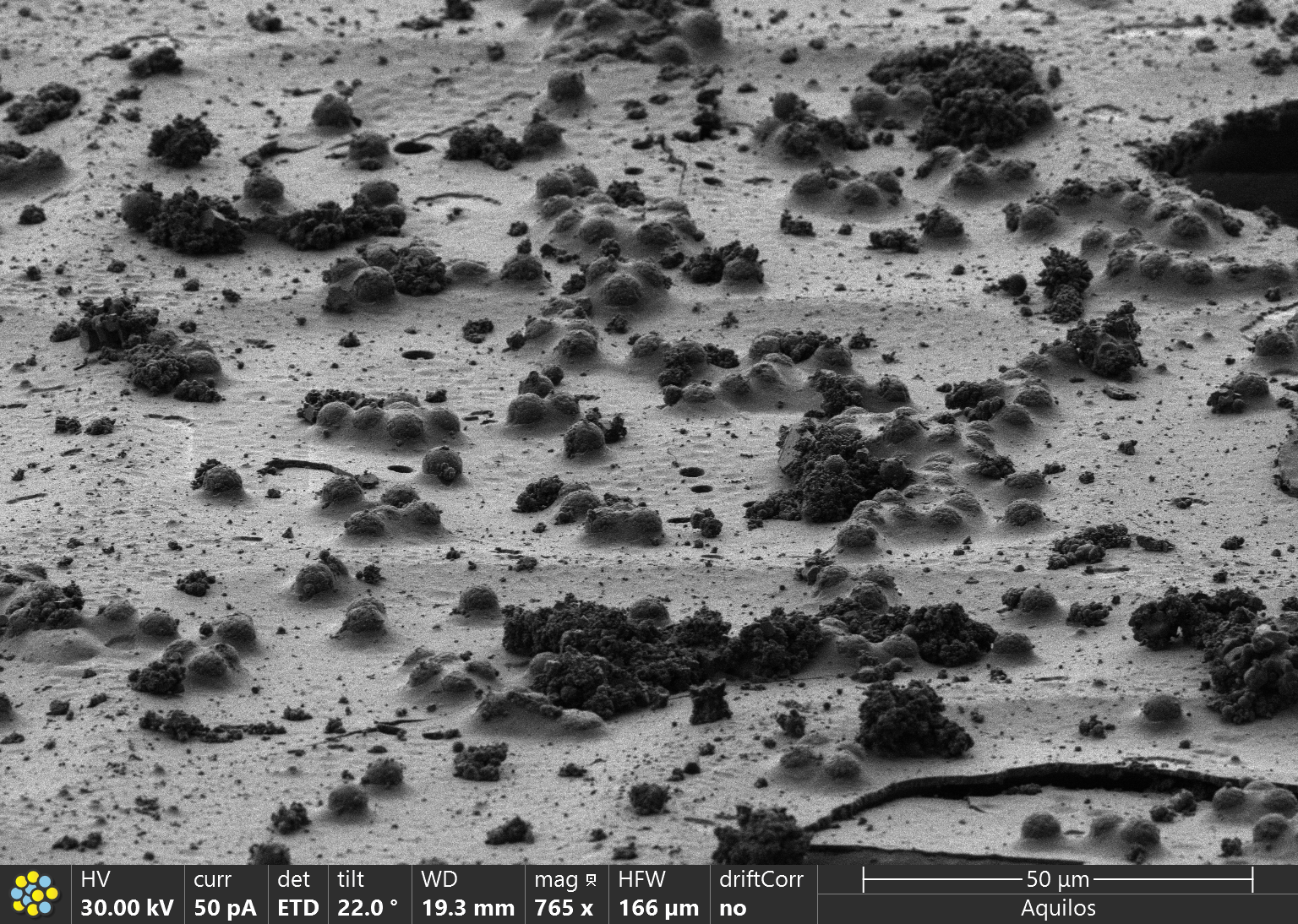

Supplement: Supplementary file 4 — Supplementary Data 1-9 [file 42003_2023_4850_MOESM4_ESM.zip › Supplementary Data 5/raw-cryoFIB-micrograph-Fig.3g.bmp]

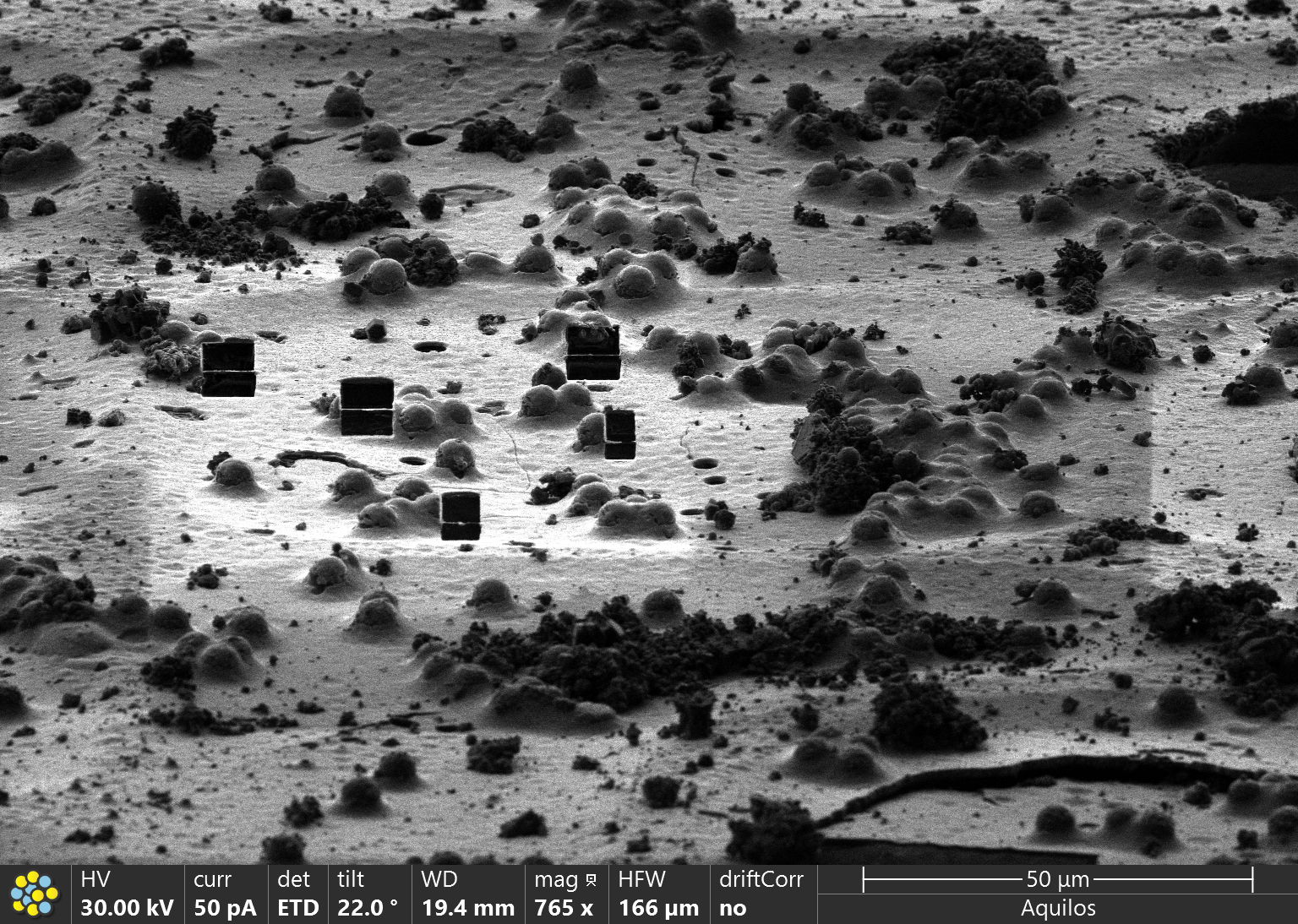

Supplement: Supplementary file 4 — Supplementary Data 1-9 [file 42003_2023_4850_MOESM4_ESM.zip › Supplementary Data 5/raw-cryoFIB-micrograph-Fig.3h.bmp]

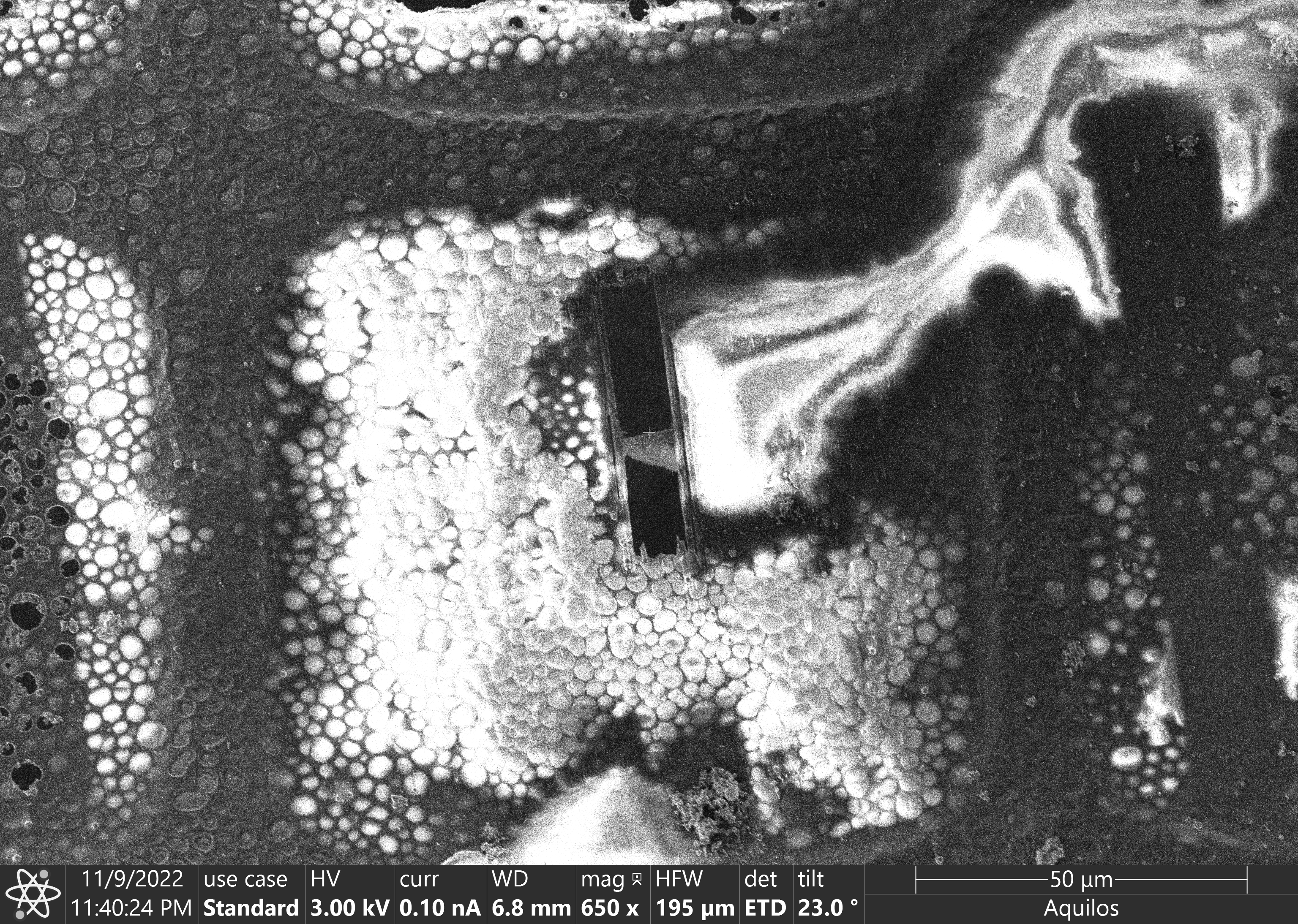

Supplement: Supplementary file 4 — Supplementary Data 1-9 [file 42003_2023_4850_MOESM4_ESM.zip › Supplementary Data 6/raw-cryoSEM-micrograph-Fig.4f.tif]

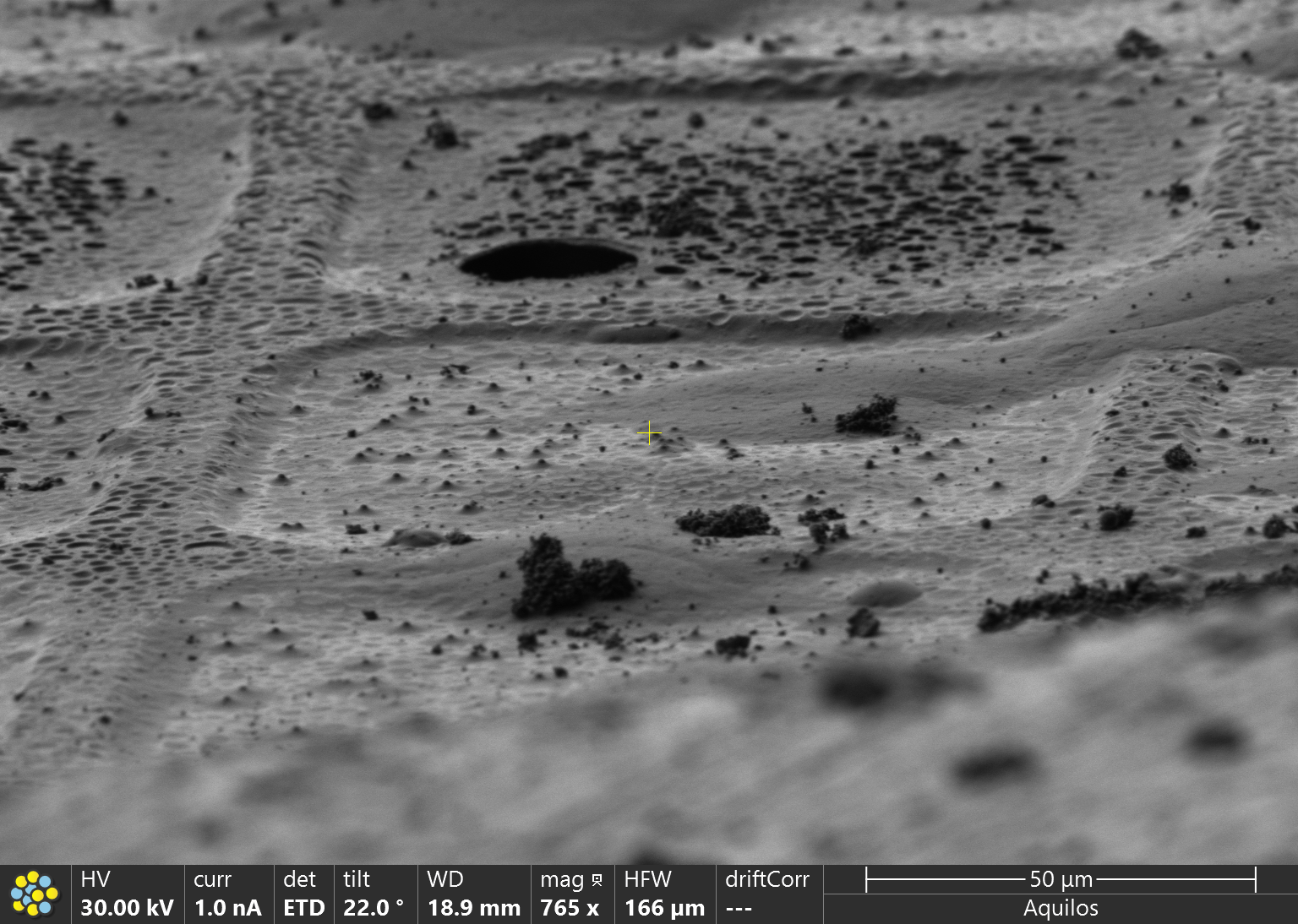

Supplement: Supplementary file 4 — Supplementary Data 1-9 [file 42003_2023_4850_MOESM4_ESM.zip › Supplementary Data 6/raw-cryoFIB-micrograph-Fig.4d.bmp]

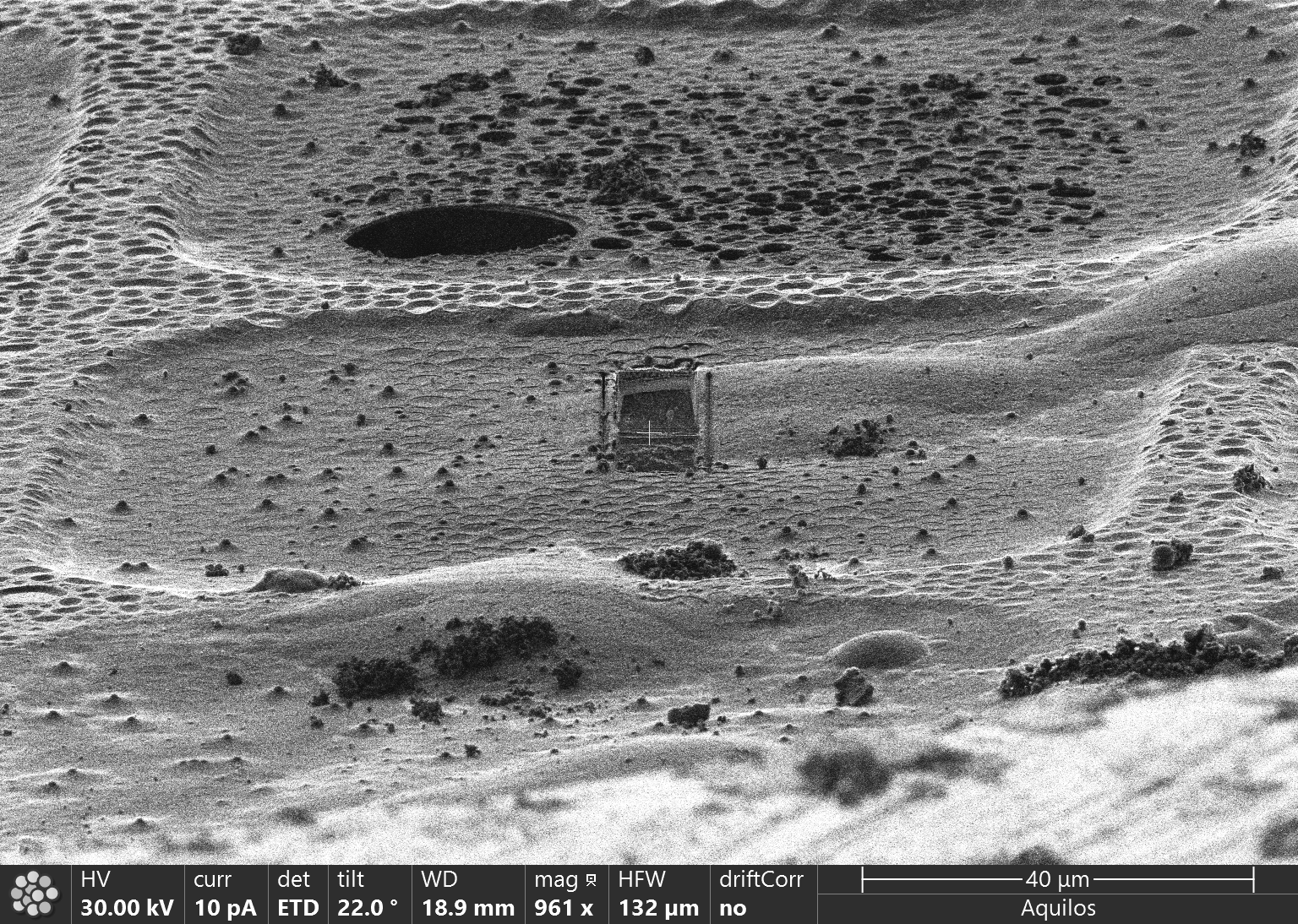

Supplement: Supplementary file 4 — Supplementary Data 1-9 [file 42003_2023_4850_MOESM4_ESM.zip › Supplementary Data 6/raw-cryoFIB-micrograph-Fig.4e.tif]

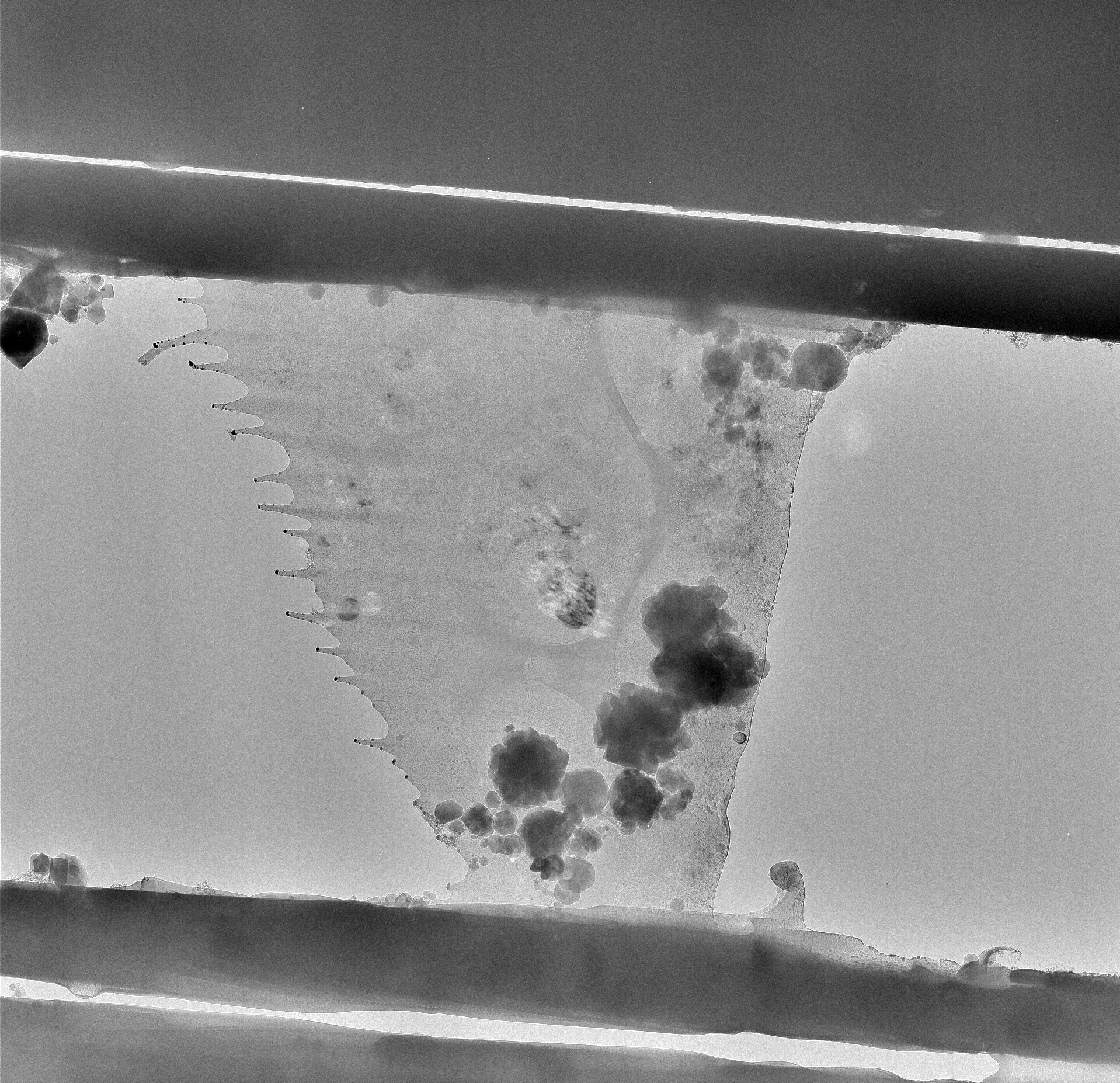

Supplement: Supplementary file 4 — Supplementary Data 1-9 [file 42003_2023_4850_MOESM4_ESM.zip › Supplementary Data 6/raw-cryoEM-micrograph-Fig.4g.jpg]

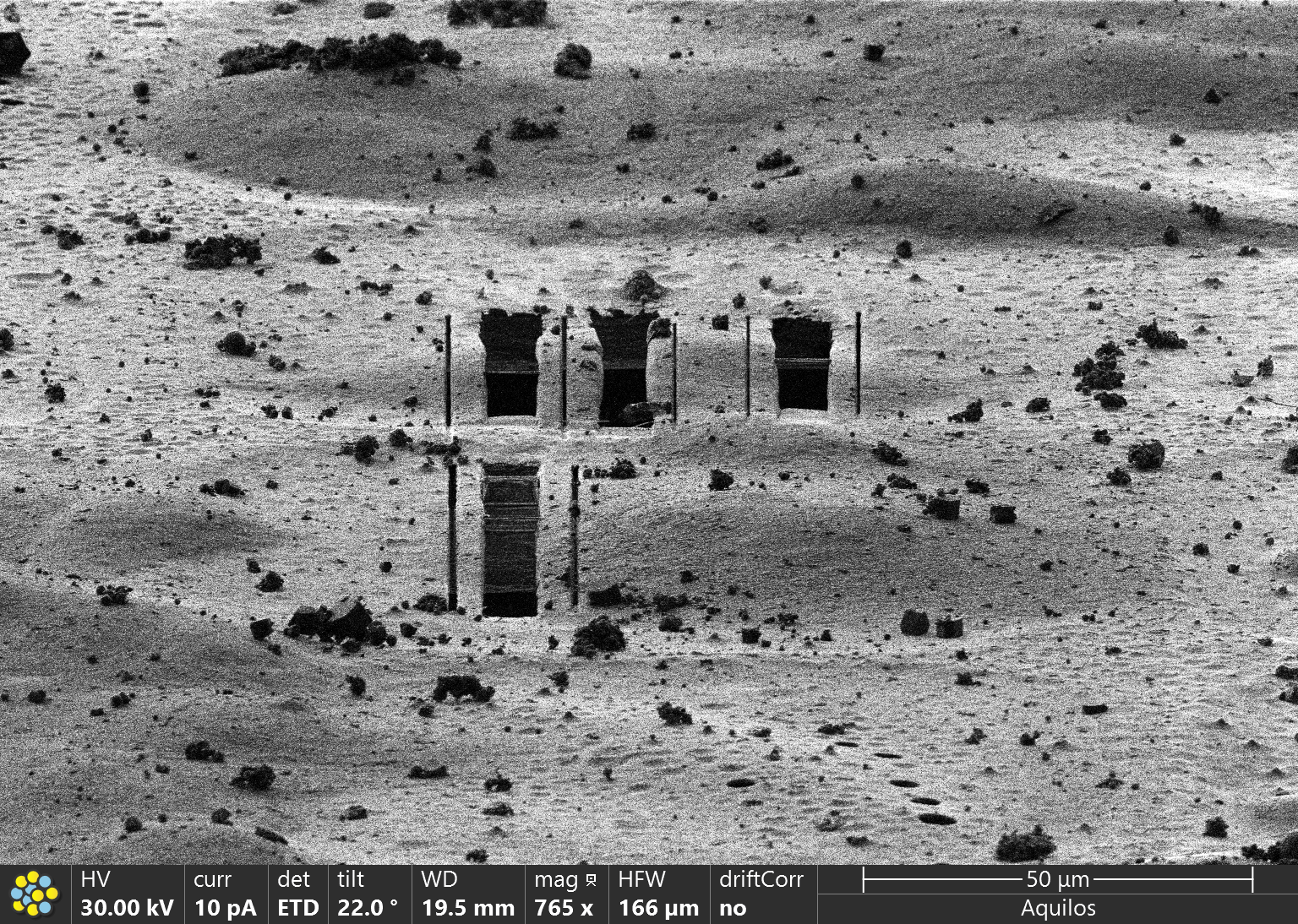

Supplement: Supplementary file 4 — Supplementary Data 1-9 [file 42003_2023_4850_MOESM4_ESM.zip › Supplementary Data 7/raw-cryoFIB-image-Fig.5e.bmp]

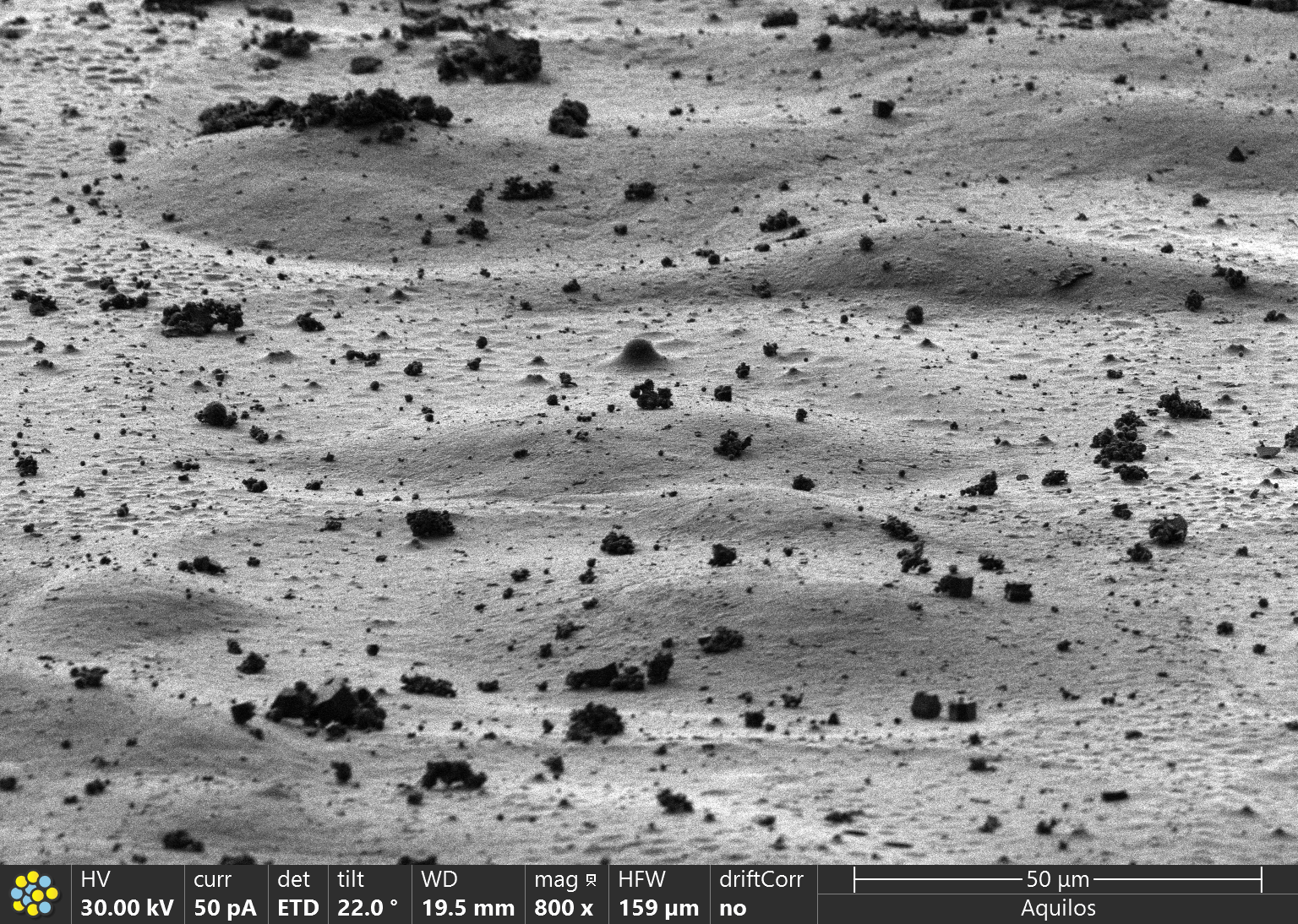

Supplement: Supplementary file 4 — Supplementary Data 1-9 [file 42003_2023_4850_MOESM4_ESM.zip › Supplementary Data 7/raw-cryoFIB-image-Fig.5d.bmp]

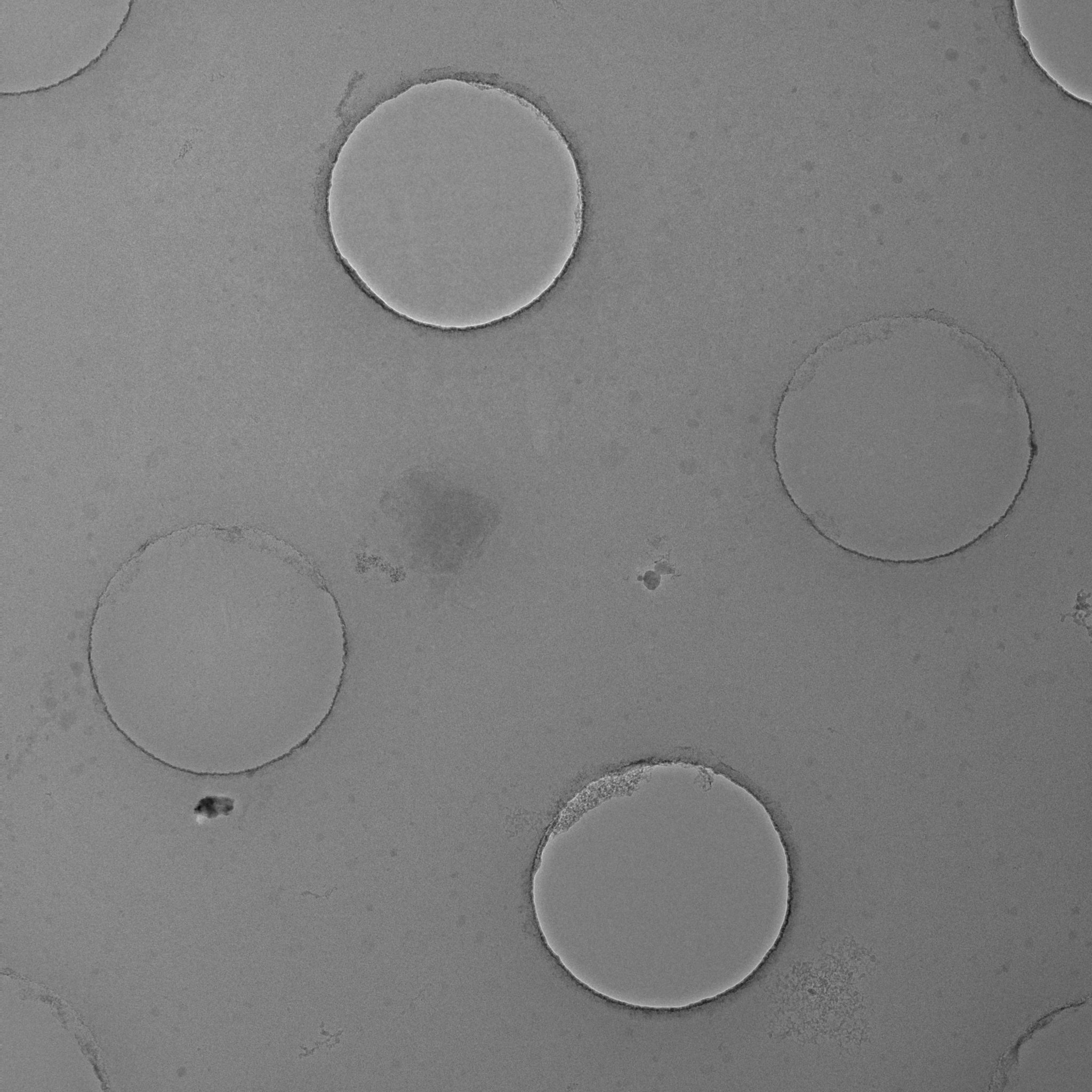

Supplement: Supplementary file 4 — Supplementary Data 1-9 [file 42003_2023_4850_MOESM4_ESM.zip › Supplementary Data 8/raw-cryoEM-micrograph-SFig.4a.jpg]

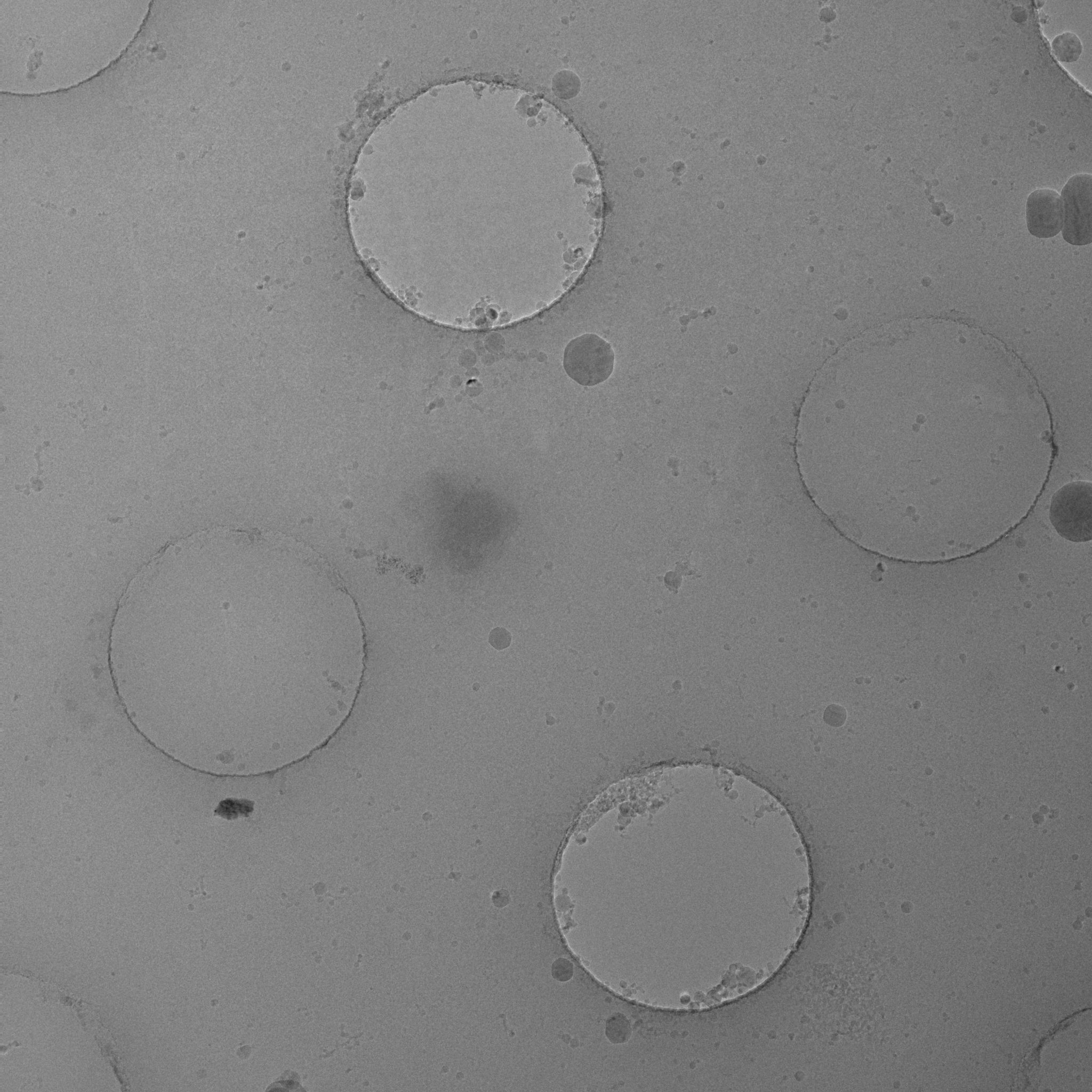

Supplement: Supplementary file 4 — Supplementary Data 1-9 [file 42003_2023_4850_MOESM4_ESM.zip › Supplementary Data 8/raw-cryoEM-micrograph-SFig.4b.jpg]

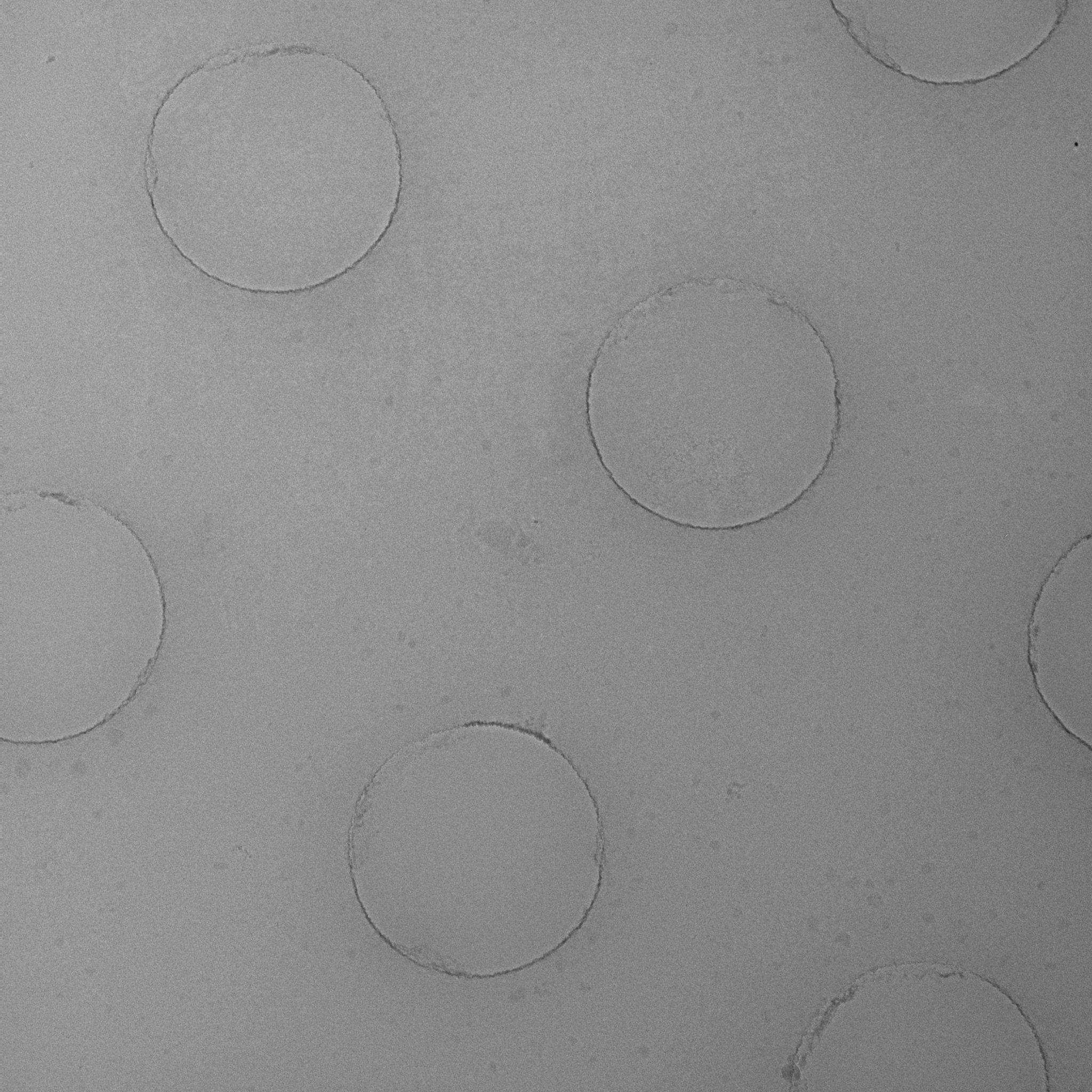

Supplement: Supplementary file 4 — Supplementary Data 1-9 [file 42003_2023_4850_MOESM4_ESM.zip › Supplementary Data 8/raw-cryoEM-micrograph-SFig.4c.jpg]

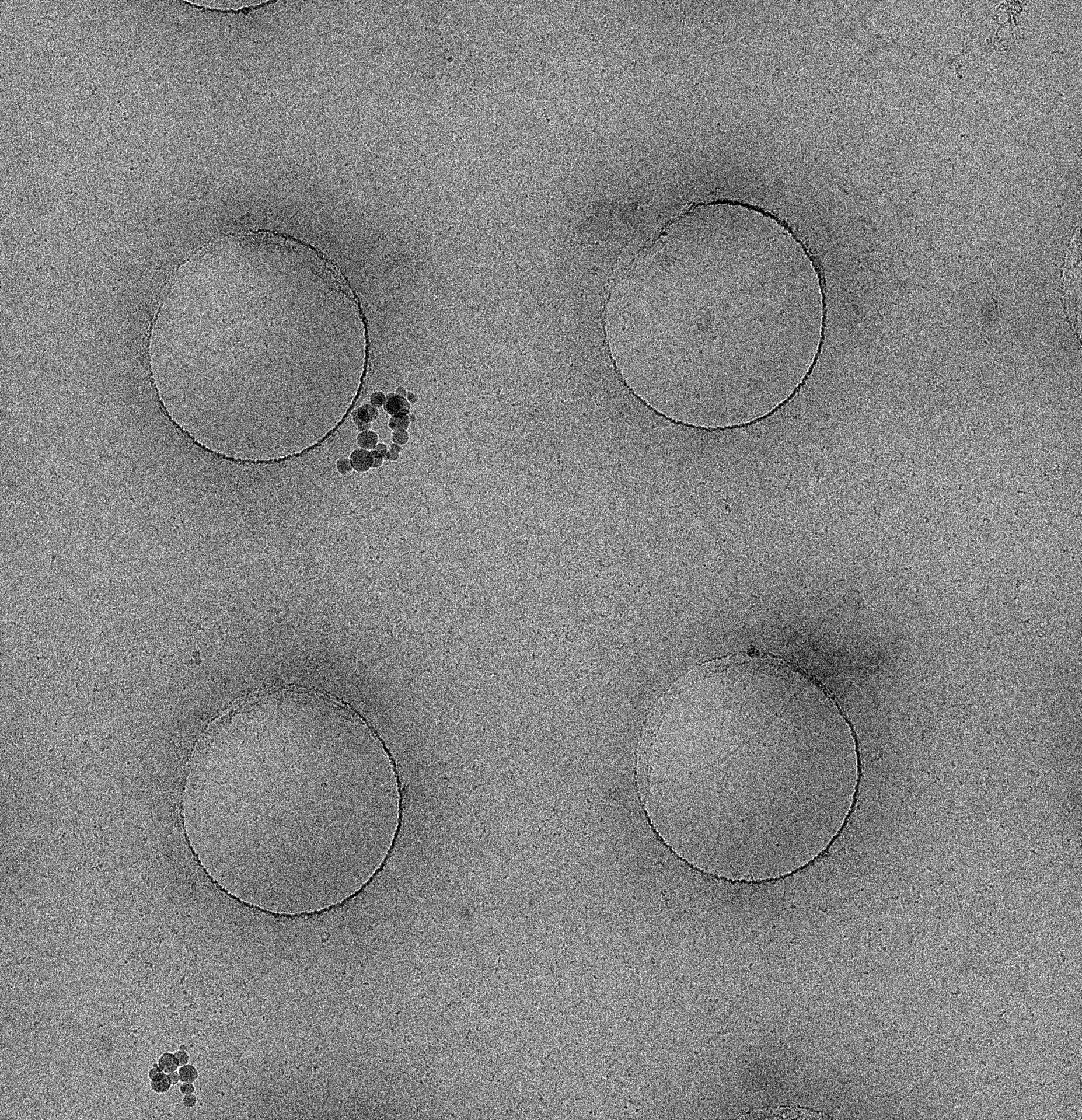

Supplement: Supplementary file 4 — Supplementary Data 1-9 [file 42003_2023_4850_MOESM4_ESM.zip › Supplementary Data 8/raw-cryoEM-micrograph-SFig.4g.jpg]

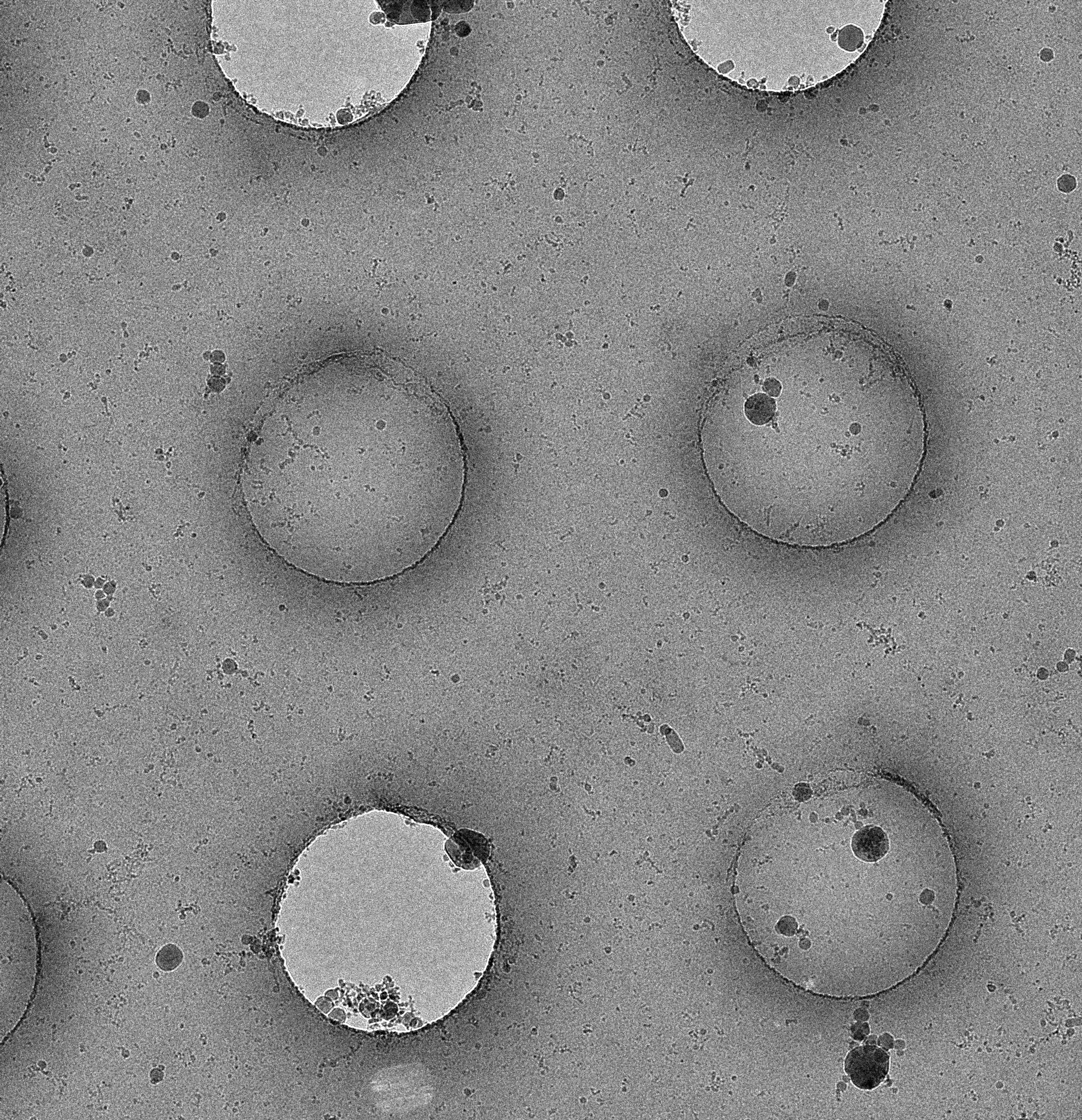

Supplement: Supplementary file 4 — Supplementary Data 1-9 [file 42003_2023_4850_MOESM4_ESM.zip › Supplementary Data 8/raw-cryoEM-micrograph-SFig.4f.jpg]

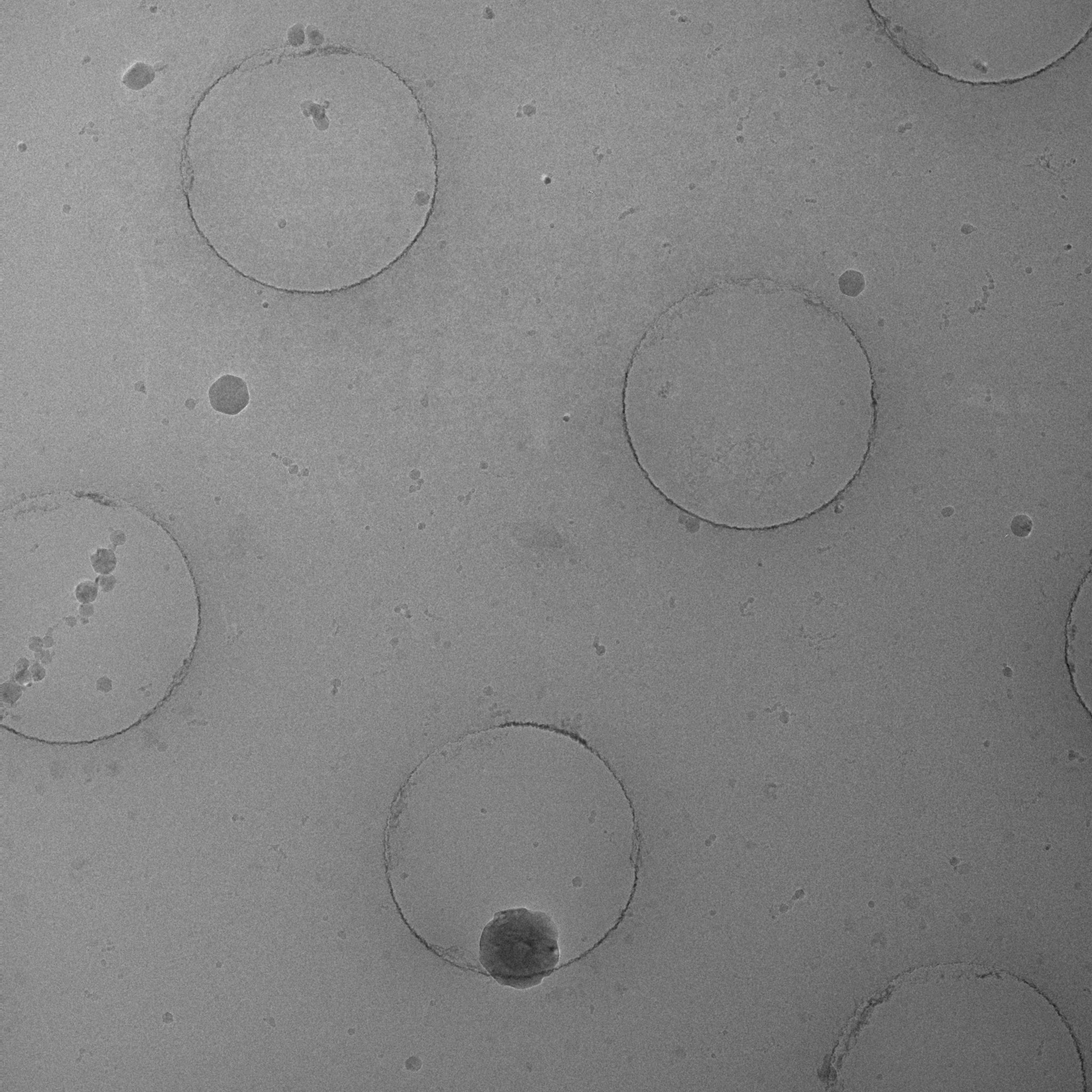

Supplement: Supplementary file 4 — Supplementary Data 1-9 [file 42003_2023_4850_MOESM4_ESM.zip › Supplementary Data 8/raw-cryoEM-micrograph-SFig.4d.jpg]

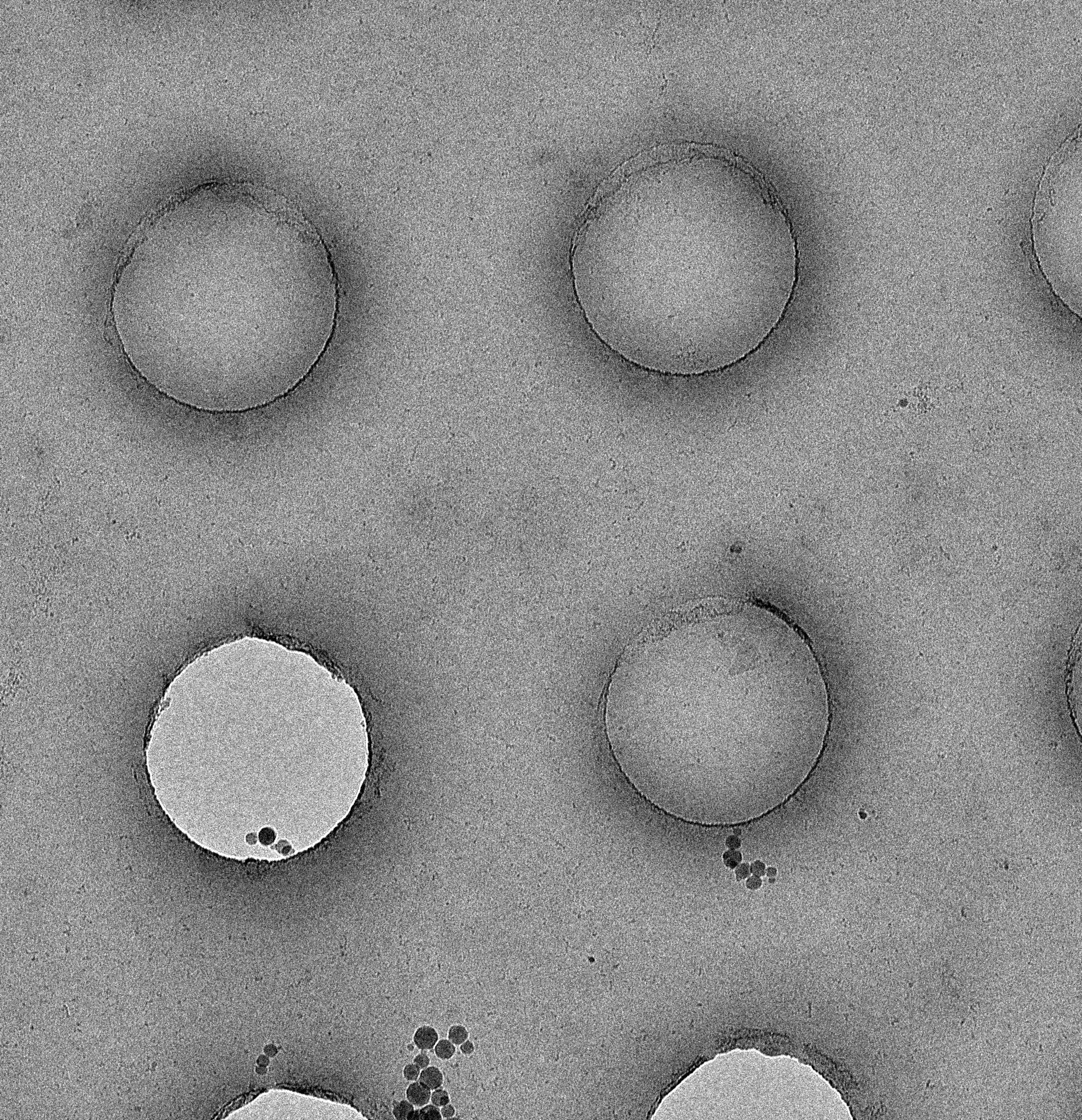

Supplement: Supplementary file 4 — Supplementary Data 1-9 [file 42003_2023_4850_MOESM4_ESM.zip › Supplementary Data 8/raw-cryoEM-micrograph-SFig.4e.jpg]

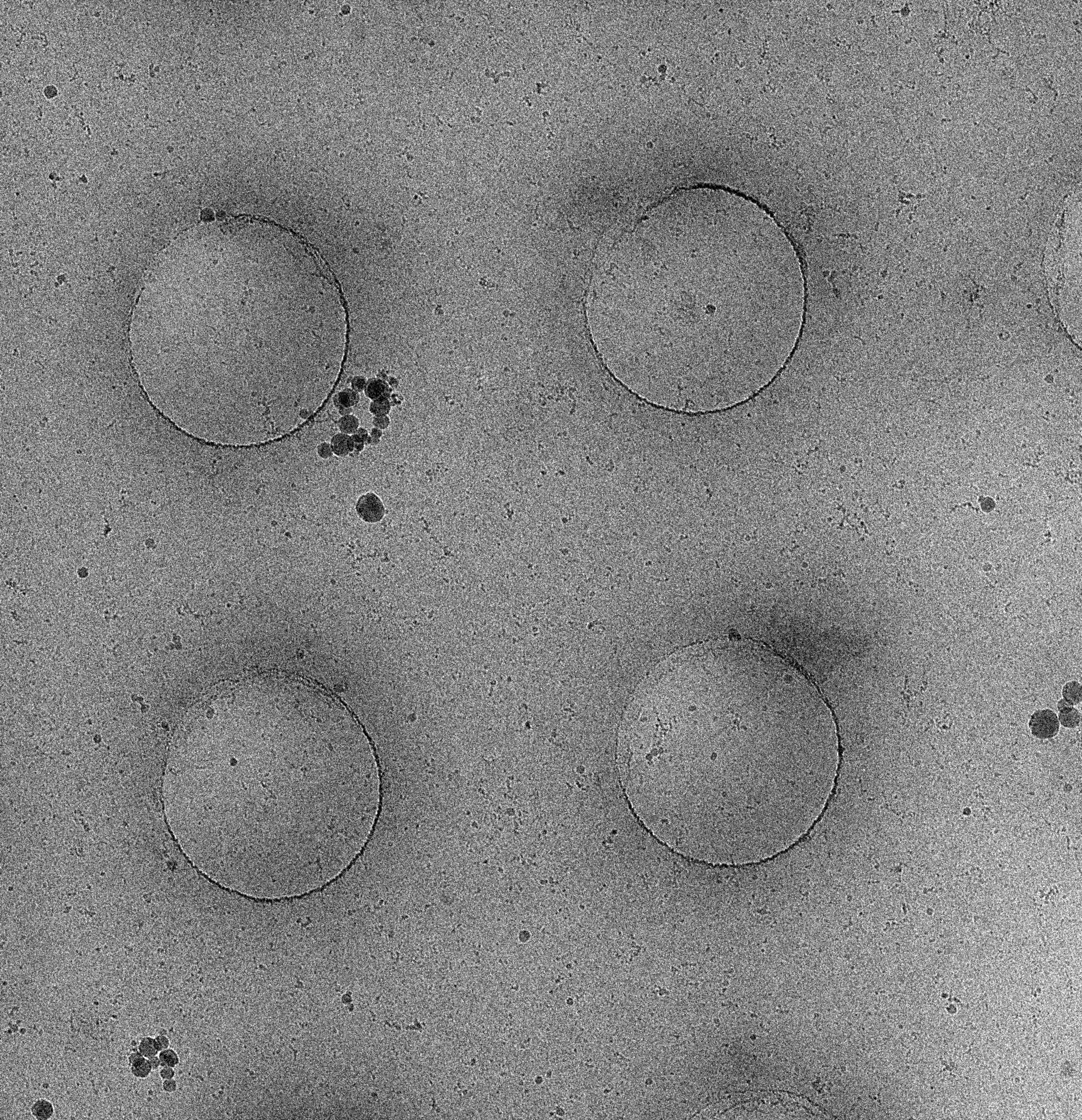

Supplement: Supplementary file 4 — Supplementary Data 1-9 [file 42003_2023_4850_MOESM4_ESM.zip › Supplementary Data 8/raw-cryoEM-micrograph-SFig.4h.jpg]

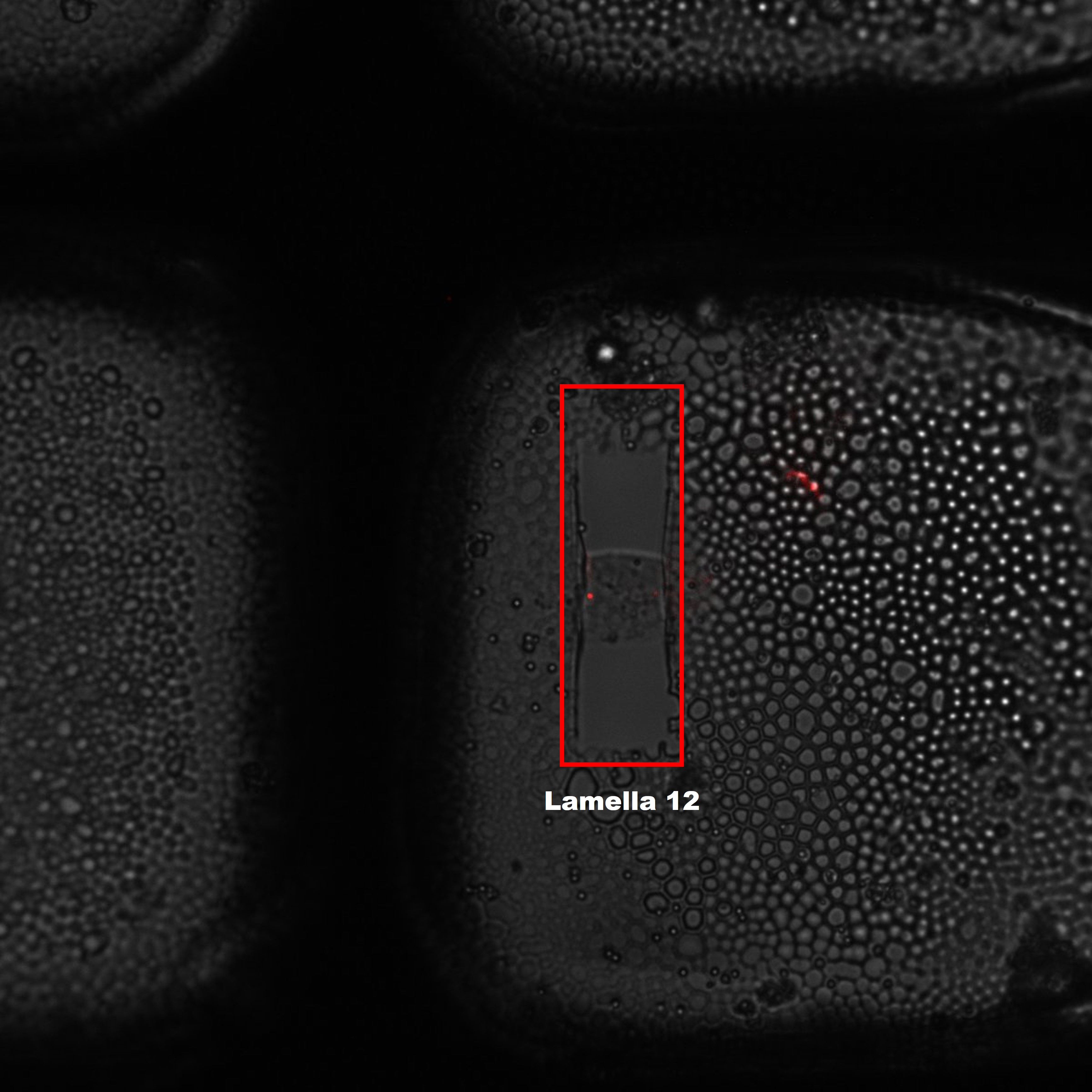

Supplement: Supplementary file 4 — Supplementary Data 1-9 [file 42003_2023_4850_MOESM4_ESM.zip › Supplementary Data 9/8.jpg]

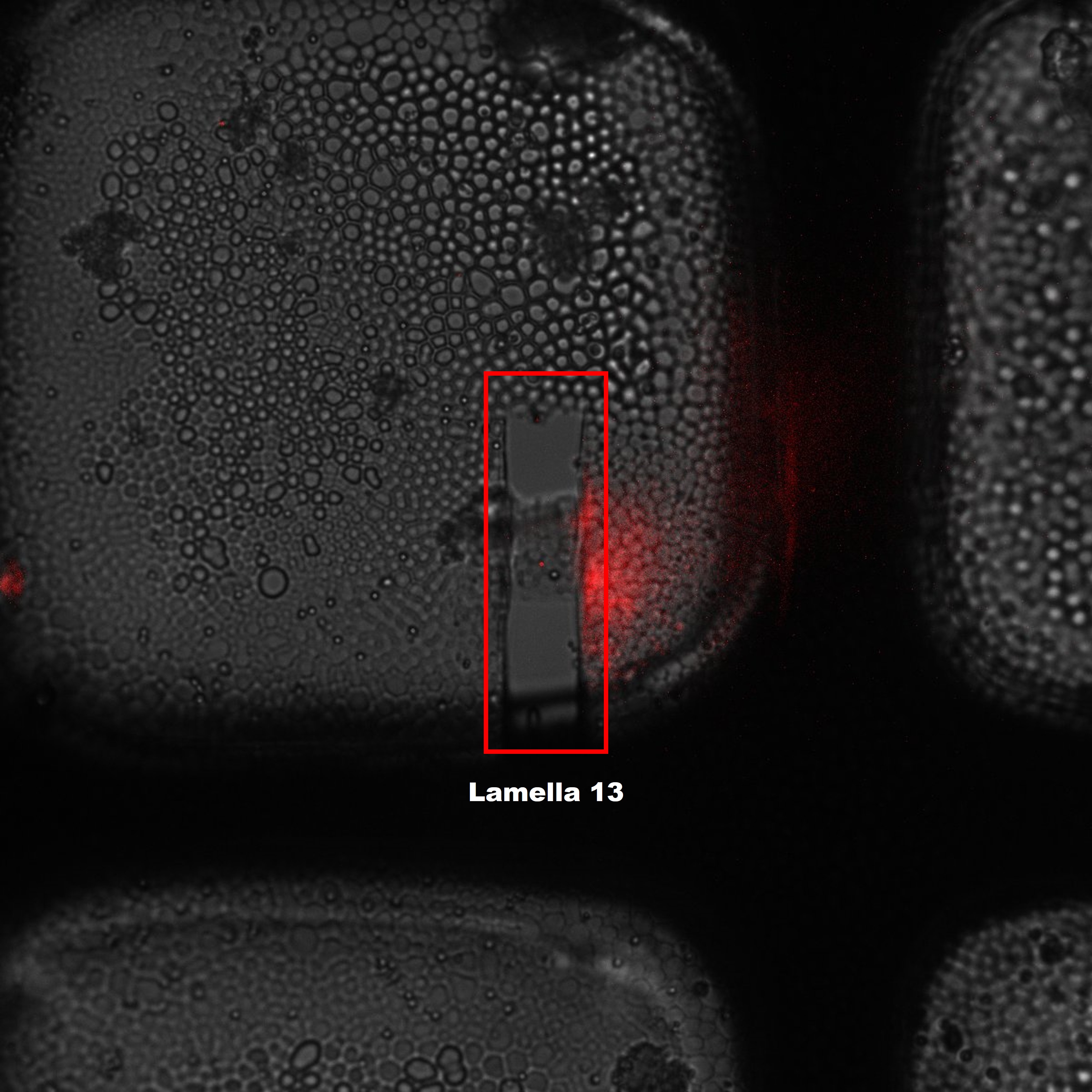

Supplement: Supplementary file 4 — Supplementary Data 1-9 [file 42003_2023_4850_MOESM4_ESM.zip › Supplementary Data 9/9.jpg]

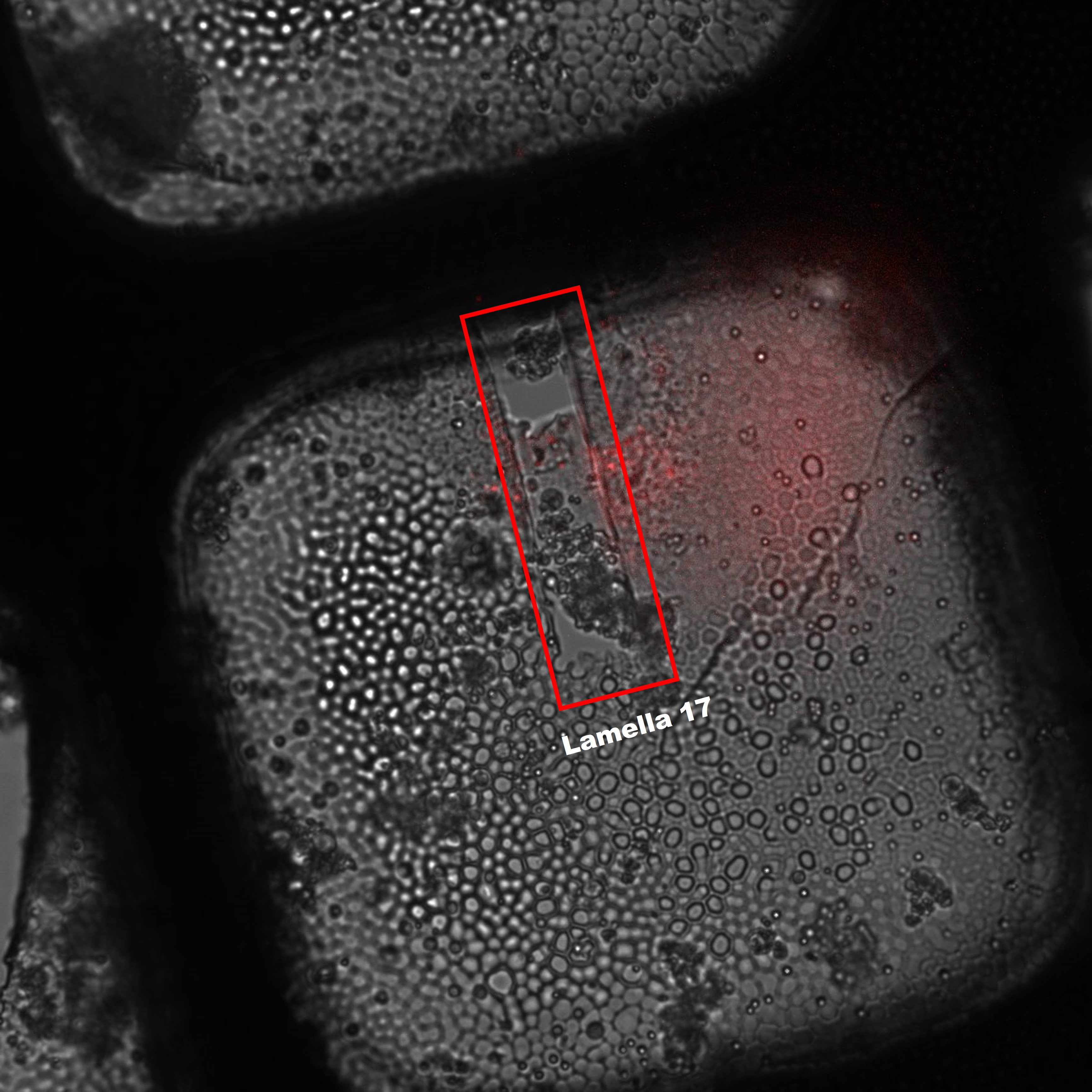

Supplement: Supplementary file 4 — Supplementary Data 1-9 [file 42003_2023_4850_MOESM4_ESM.zip › Supplementary Data 9/12.jpg]

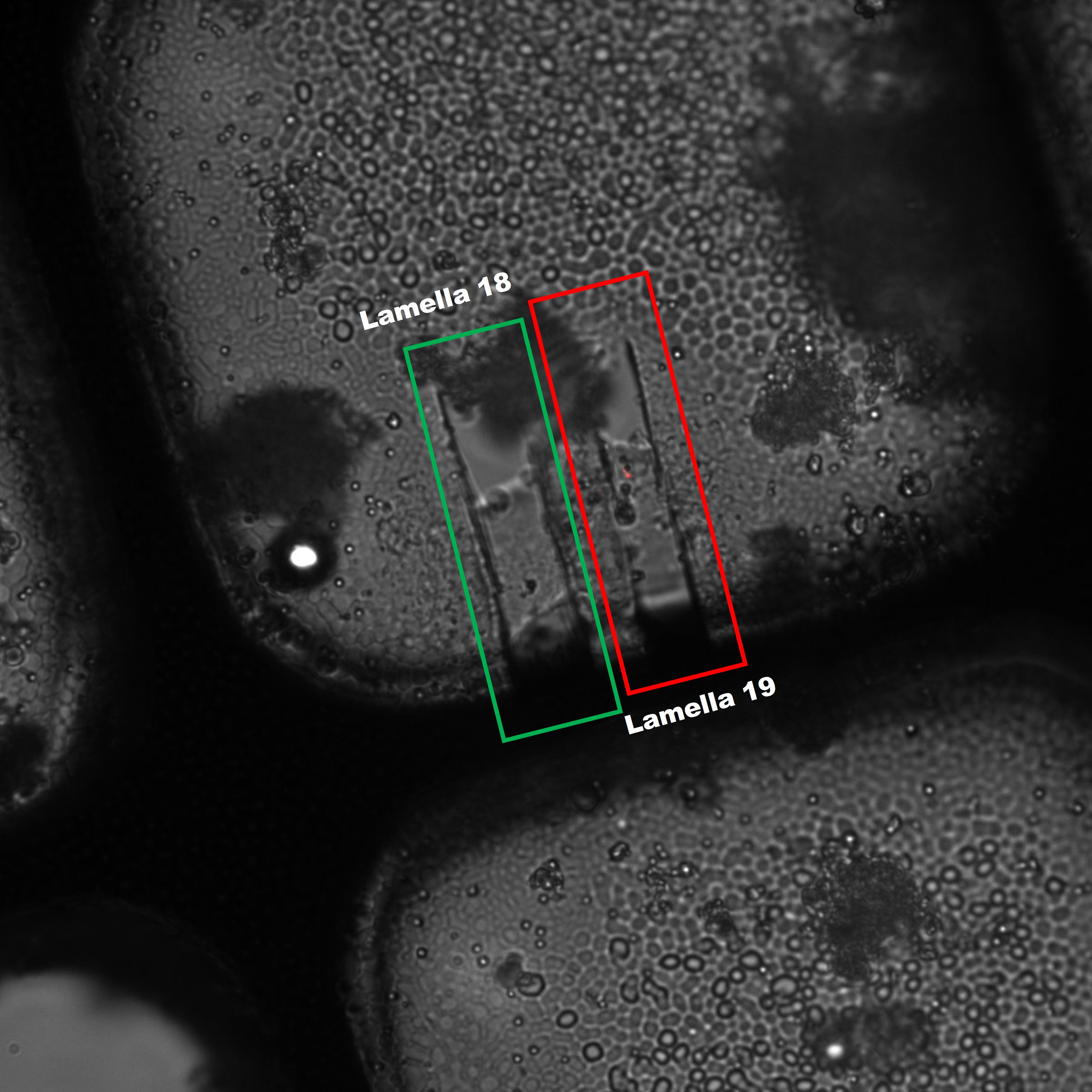

Supplement: Supplementary file 4 — Supplementary Data 1-9 [file 42003_2023_4850_MOESM4_ESM.zip › Supplementary Data 9/13.jpg]

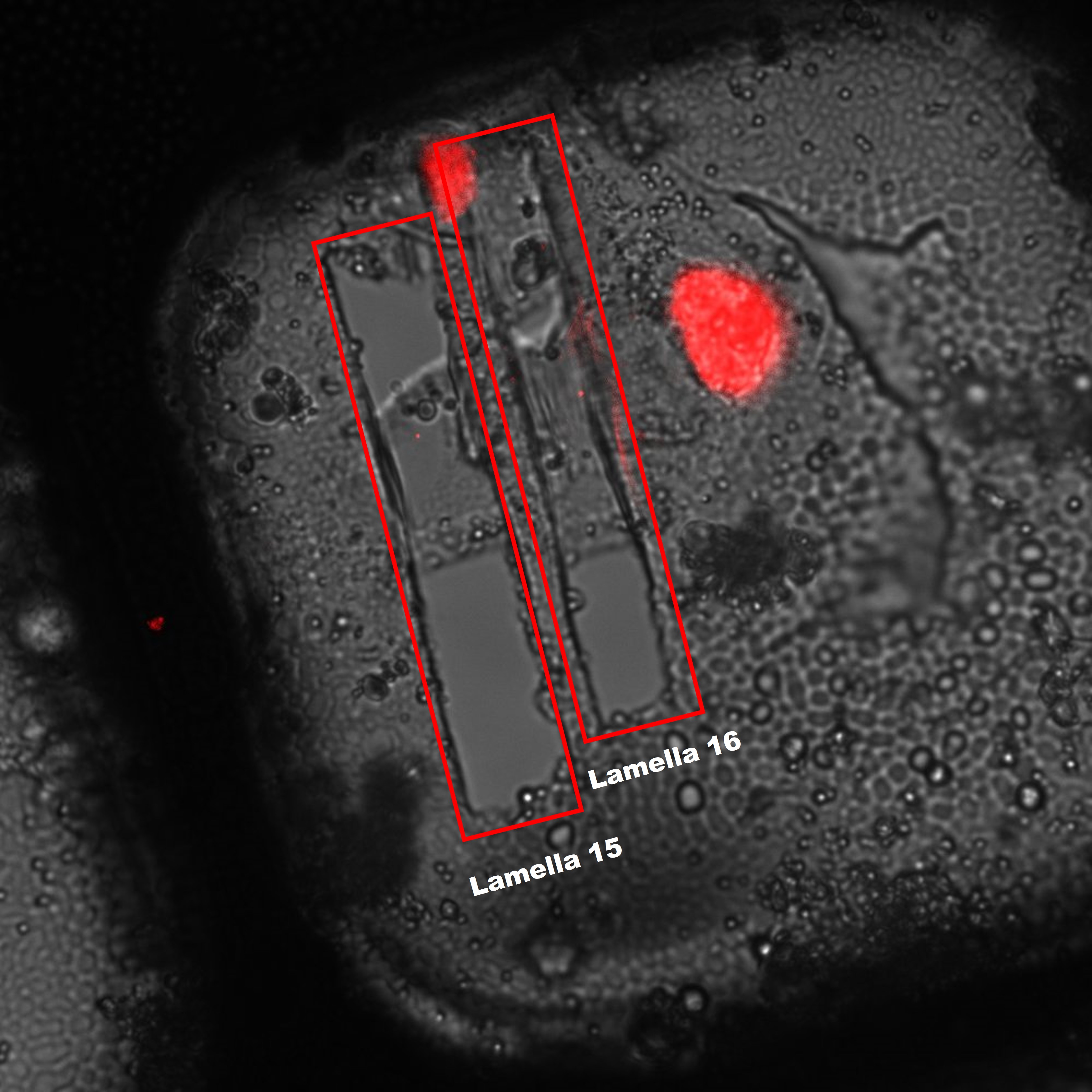

Supplement: Supplementary file 4 — Supplementary Data 1-9 [file 42003_2023_4850_MOESM4_ESM.zip › Supplementary Data 9/11.jpg]

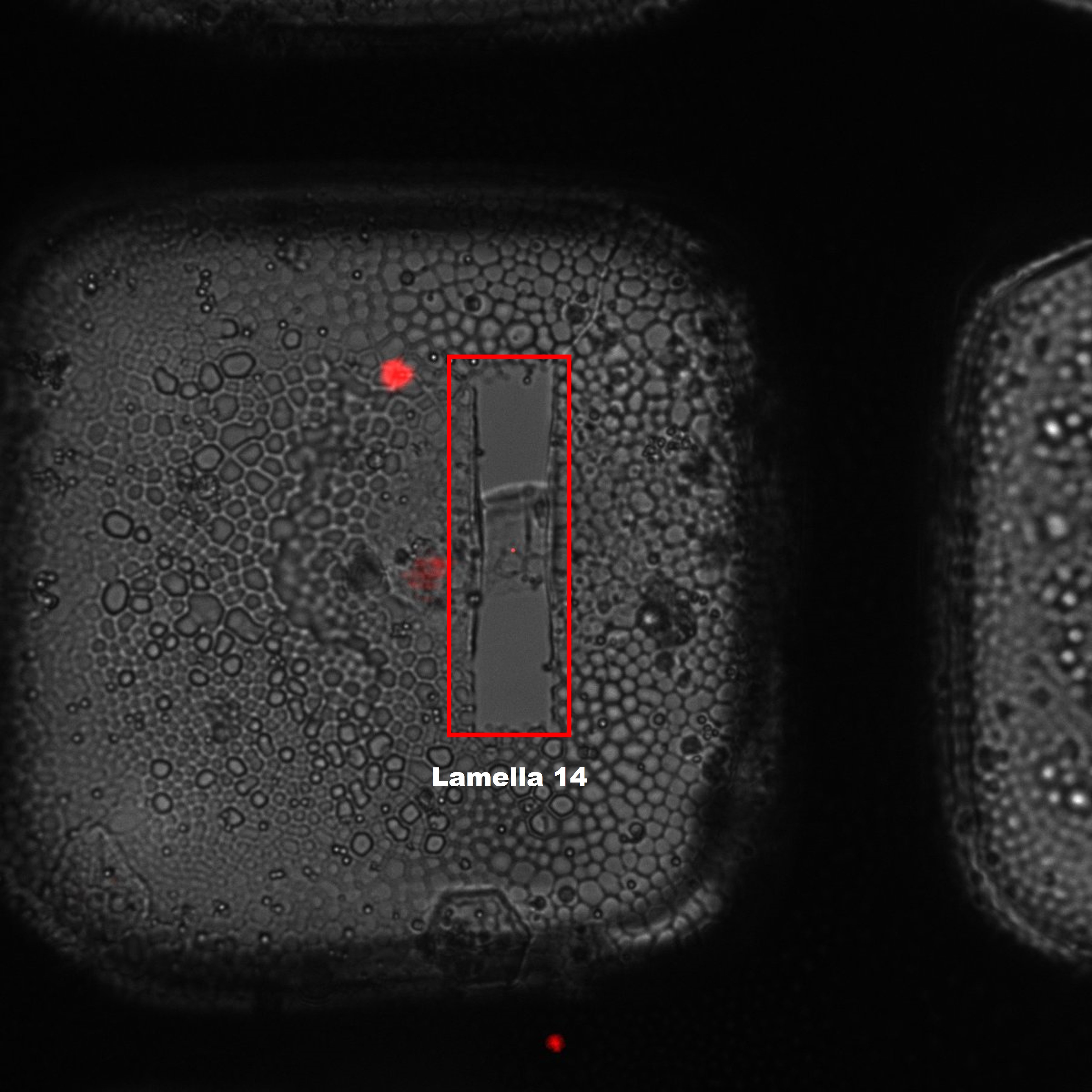

Supplement: Supplementary file 4 — Supplementary Data 1-9 [file 42003_2023_4850_MOESM4_ESM.zip › Supplementary Data 9/10.jpg]

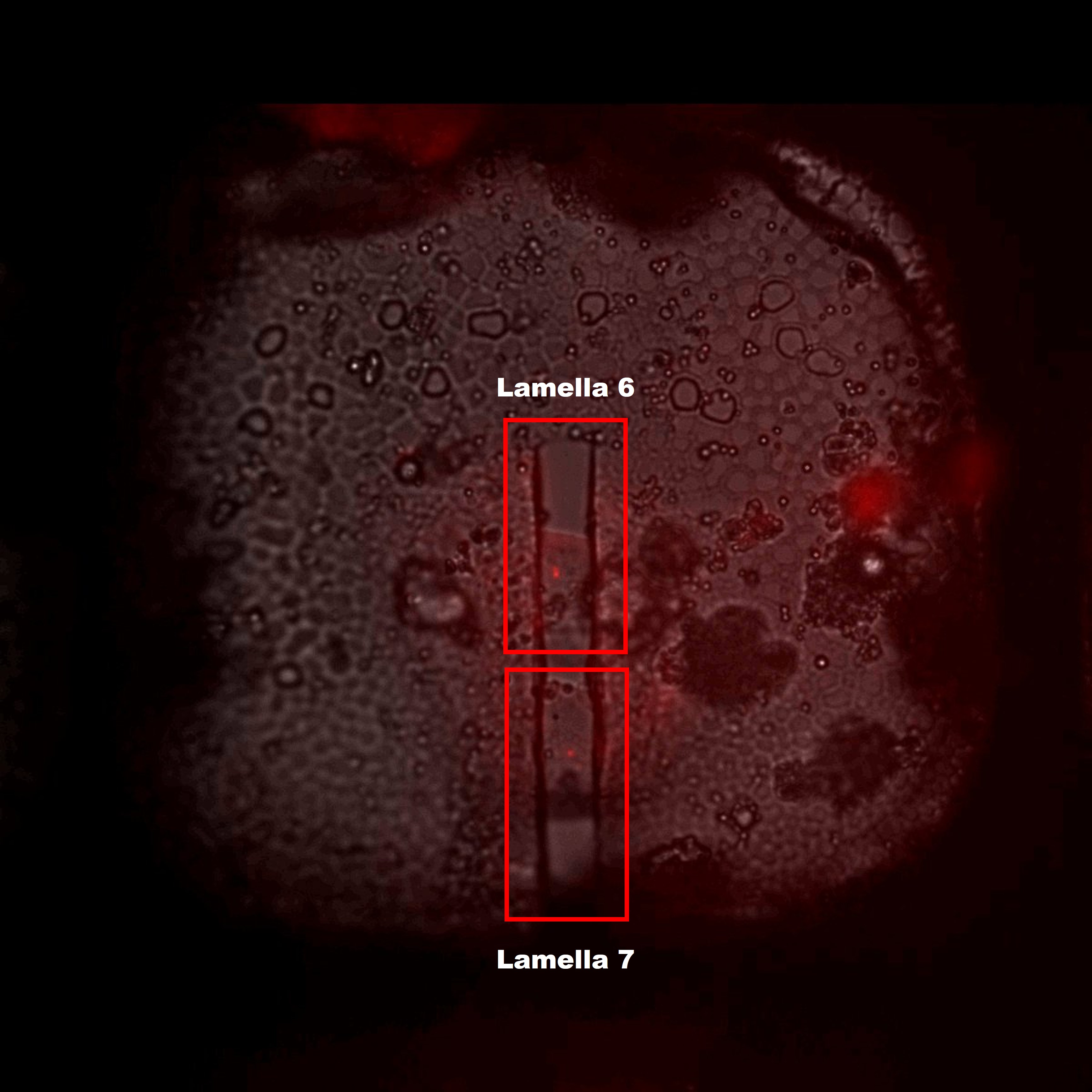

Supplement: Supplementary file 4 — Supplementary Data 1-9 [file 42003_2023_4850_MOESM4_ESM.zip › Supplementary Data 9/4.jpg]

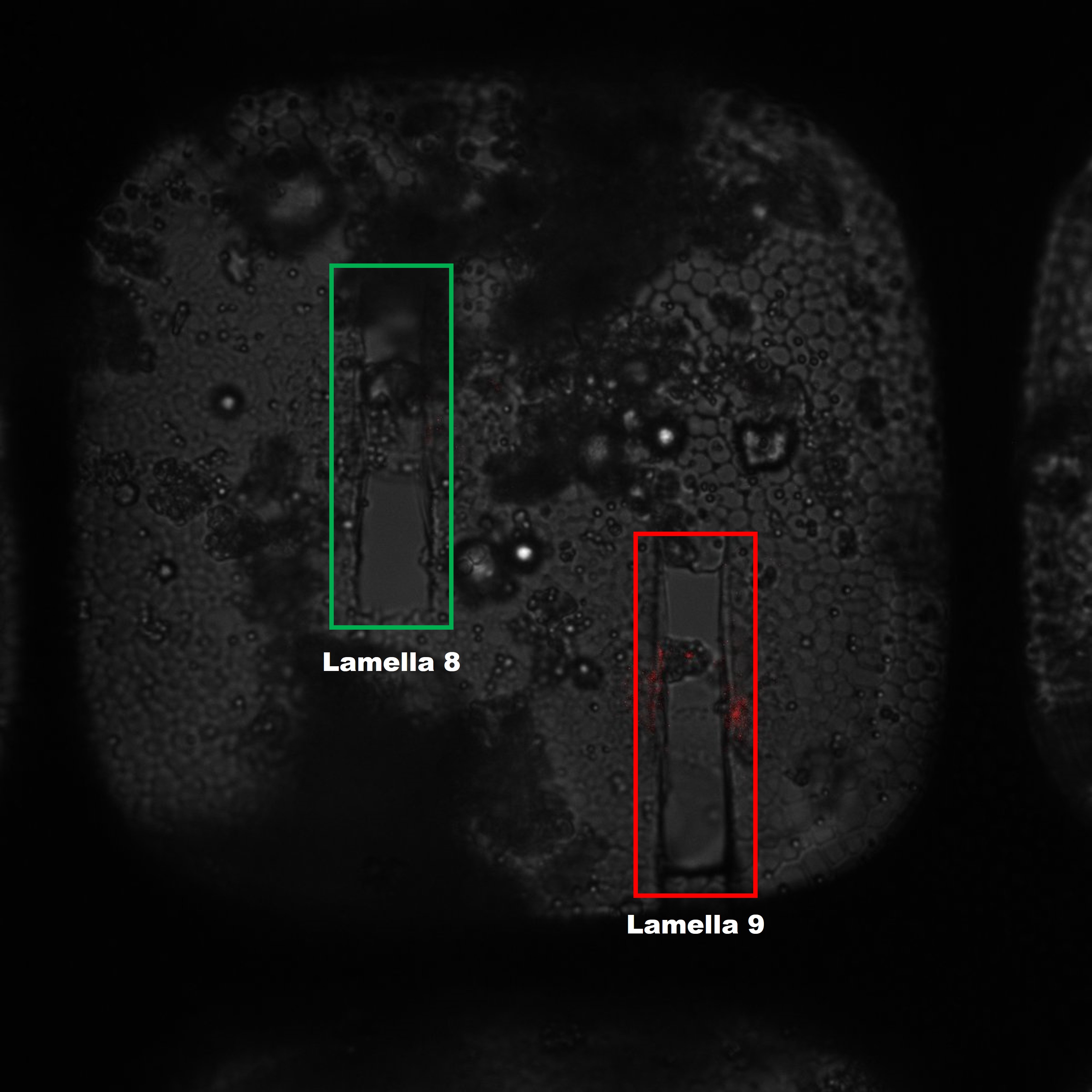

Supplement: Supplementary file 4 — Supplementary Data 1-9 [file 42003_2023_4850_MOESM4_ESM.zip › Supplementary Data 9/5.jpg]

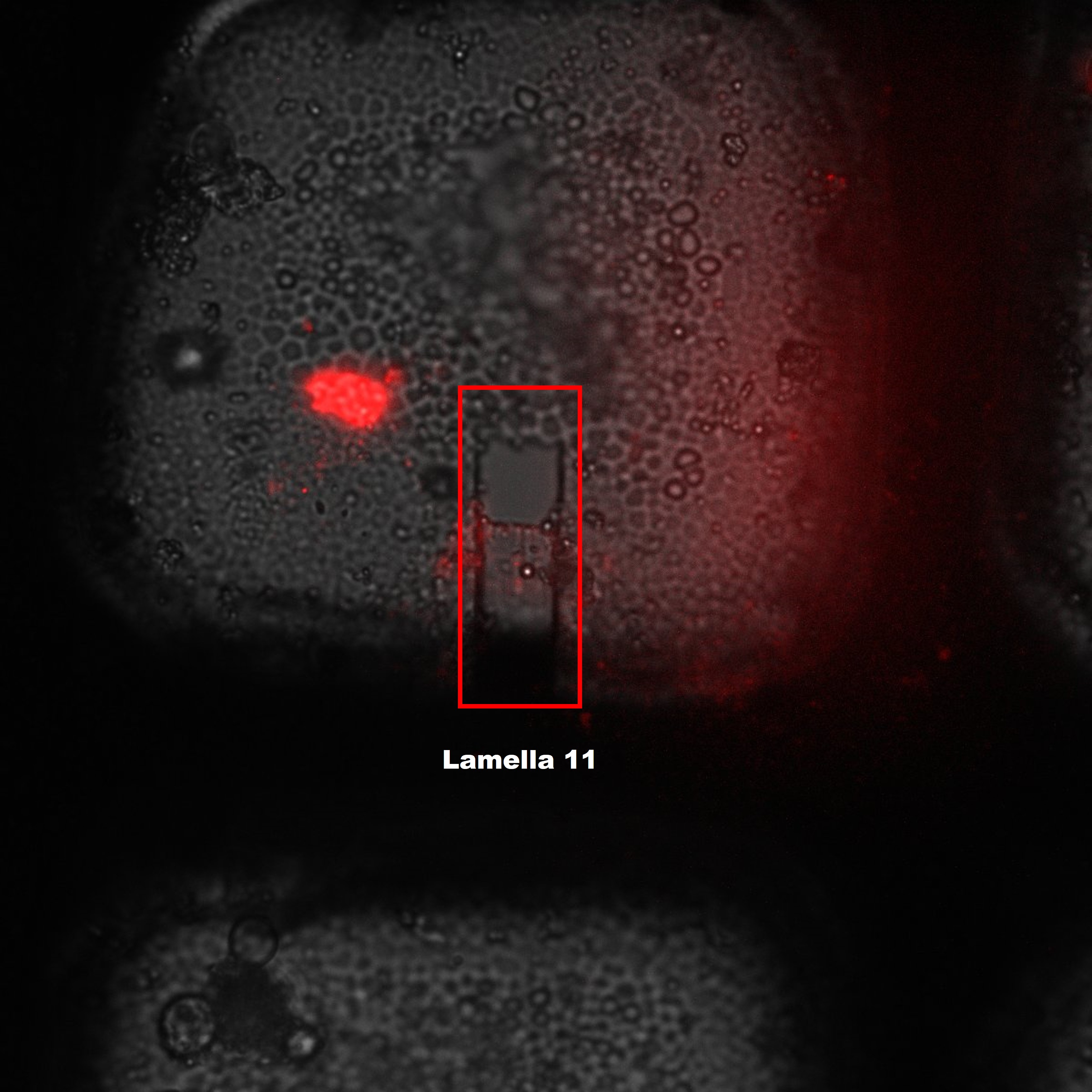

Supplement: Supplementary file 4 — Supplementary Data 1-9 [file 42003_2023_4850_MOESM4_ESM.zip › Supplementary Data 9/7.jpg]

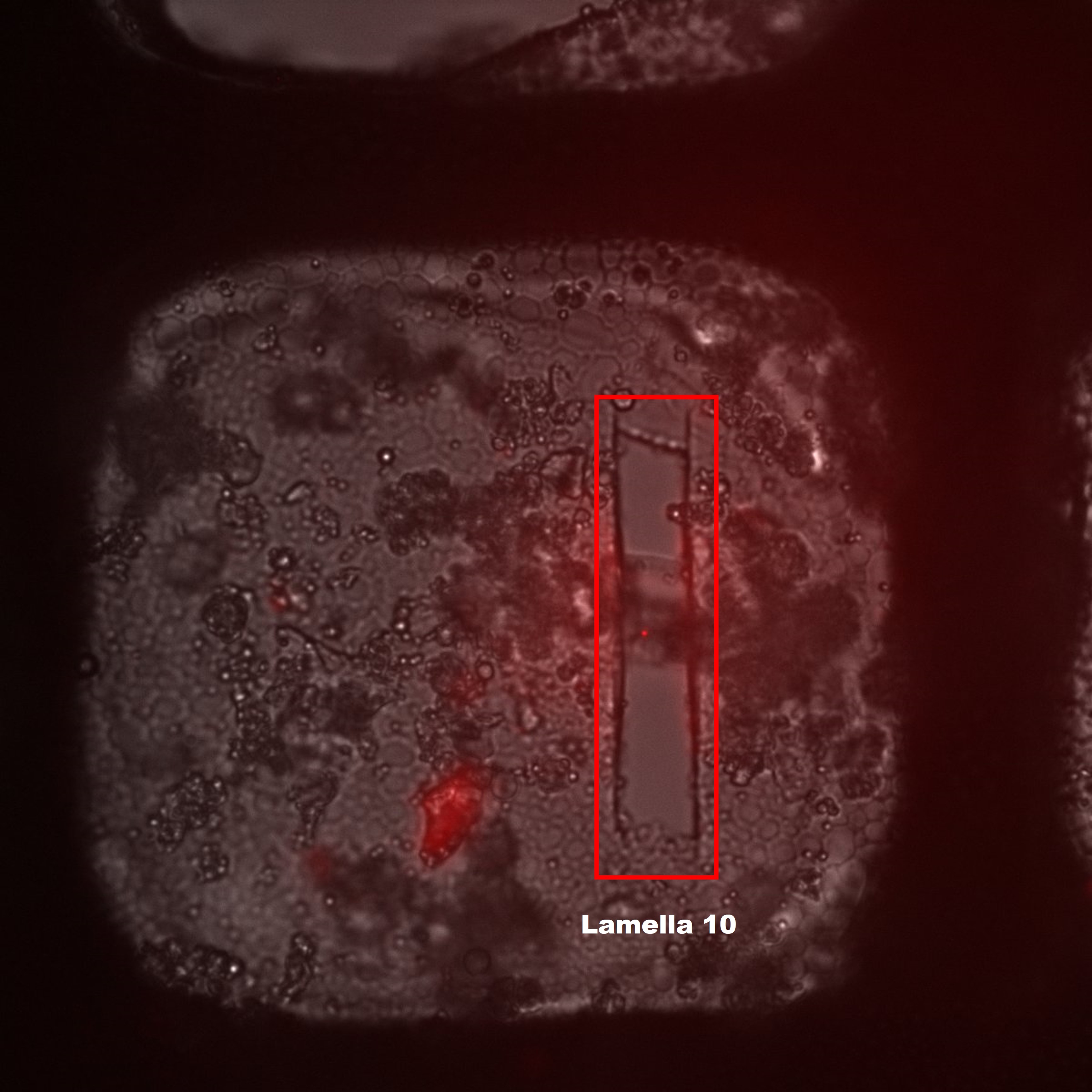

Supplement: Supplementary file 4 — Supplementary Data 1-9 [file 42003_2023_4850_MOESM4_ESM.zip › Supplementary Data 9/6.jpg]

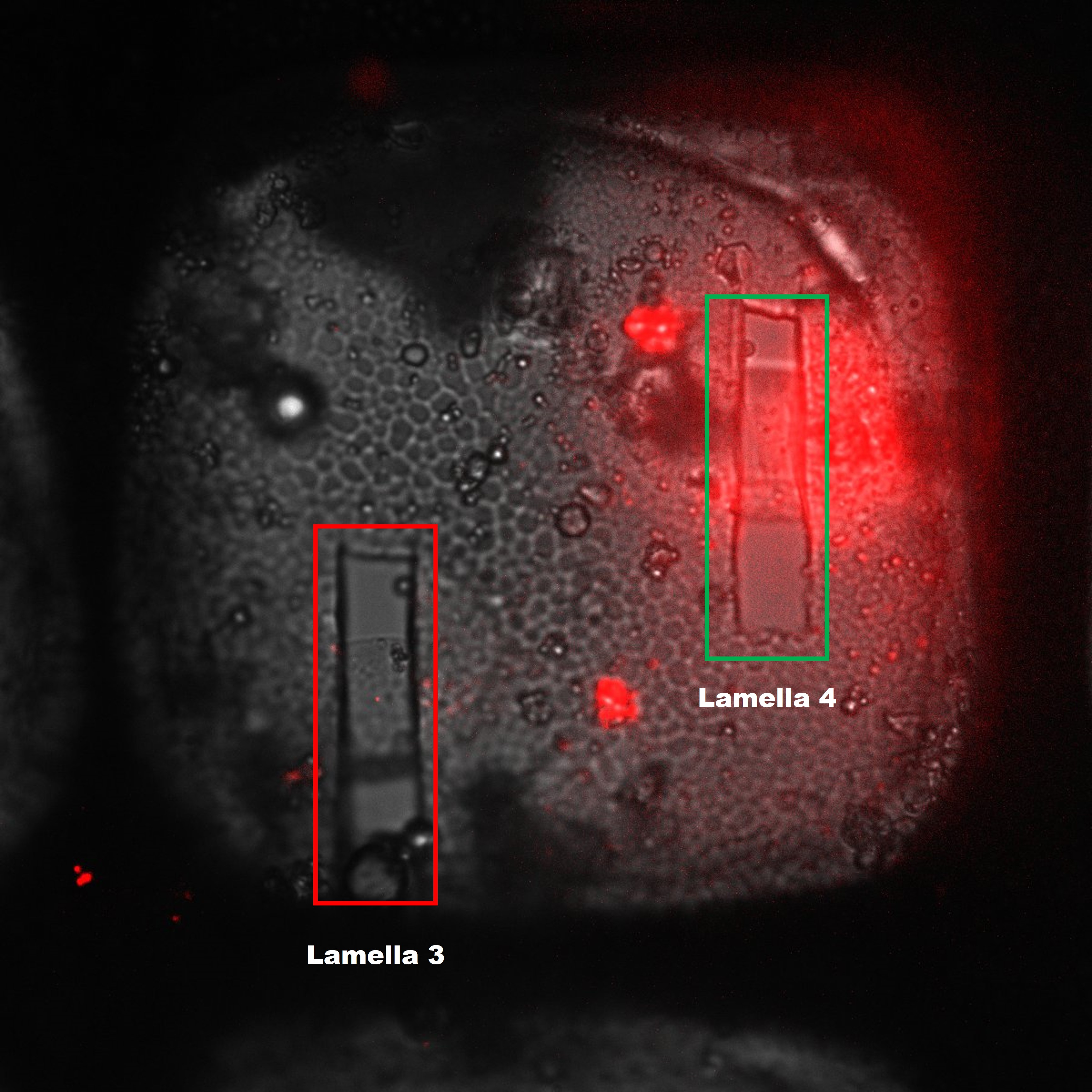

Supplement: Supplementary file 4 — Supplementary Data 1-9 [file 42003_2023_4850_MOESM4_ESM.zip › Supplementary Data 9/2.jpg]

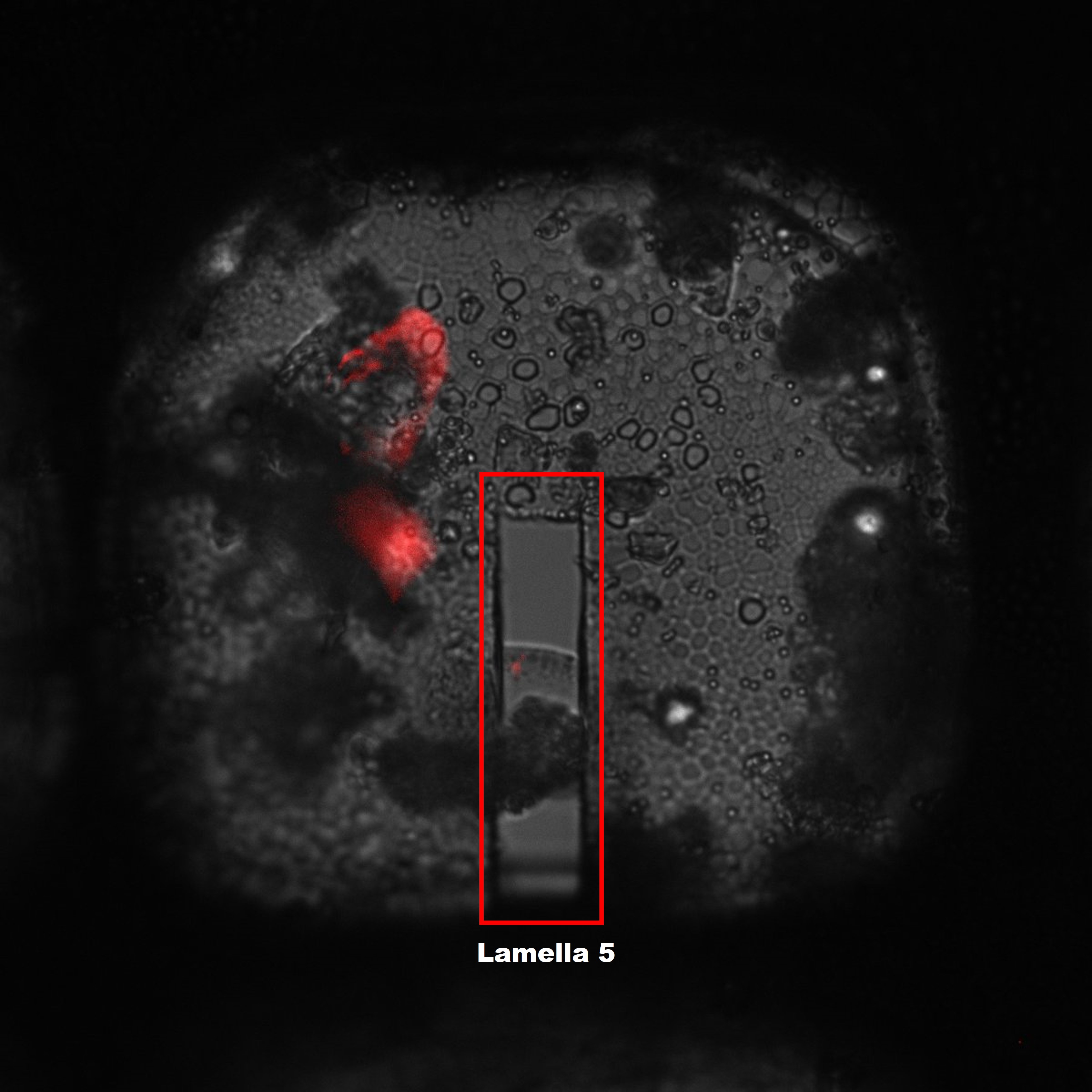

Supplement: Supplementary file 4 — Supplementary Data 1-9 [file 42003_2023_4850_MOESM4_ESM.zip › Supplementary Data 9/3.jpg]

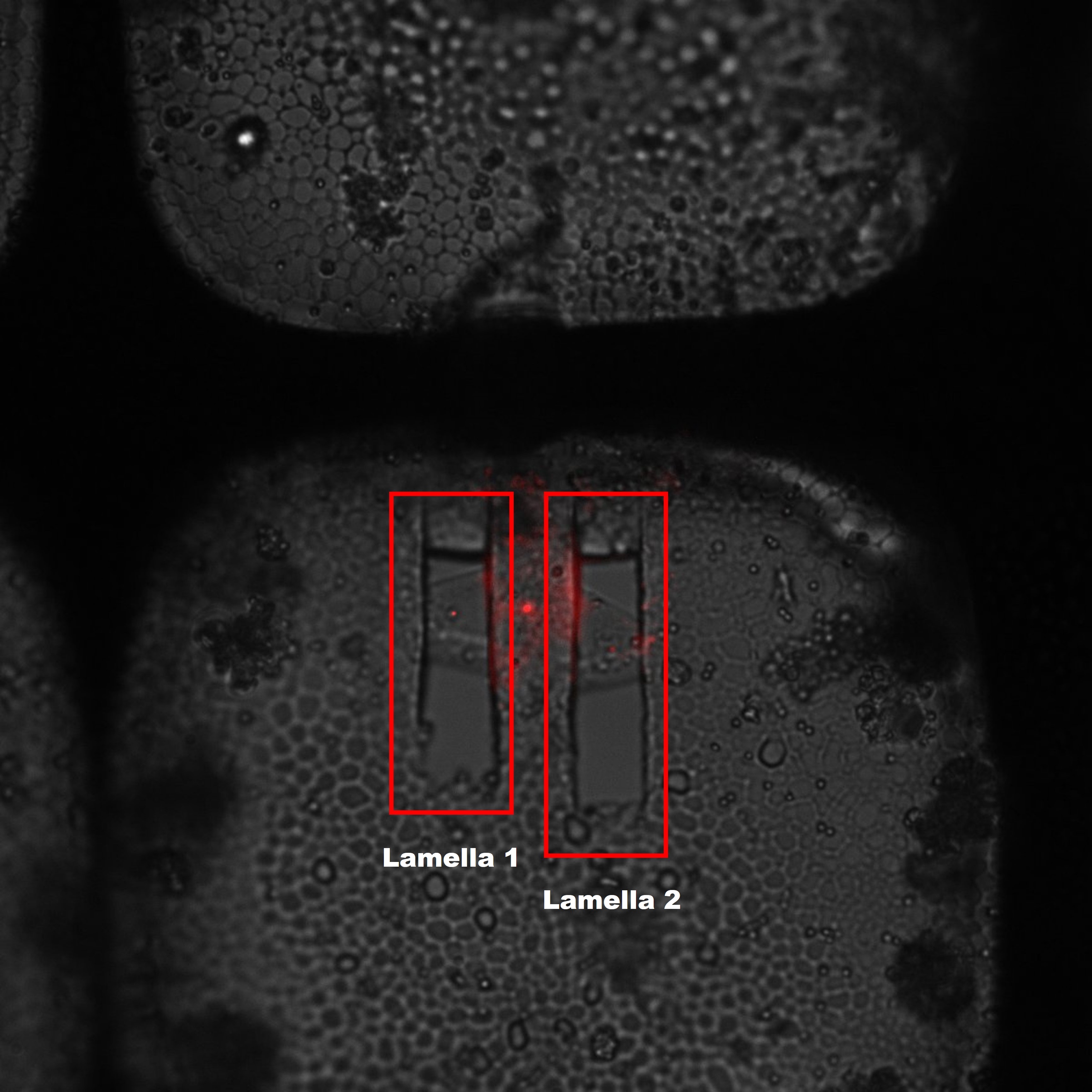

Supplement: Supplementary file 4 — Supplementary Data 1-9 [file 42003_2023_4850_MOESM4_ESM.zip › Supplementary Data 9/1.jpg]
